# Arf1 Ablation in Colorectal Cancer Cells Activates a Super Signal Complex in DC to Enhance Anti‐Tumor Immunity

**DOI:** 10.1002/advs.202305089

**Published:** 2023-10-02

**Authors:** Handong Ma, Wanqi Fang, Qiaoming Li, Yuetong Wang, Steven X. Hou

**Affiliations:** ^1^ Department of Cell and Developmental Biology at School of Life Sciences State Key Laboratory of Genetic Engineering Institute of Metabolism and Integrative Biology Human Phenome Institute Department of Liver Surgery and Transplantation of Liver Cancer Institute at Zhongshan Hospital Fudan University Shanghai 200438 China

**Keywords:** anti‐tumor immunity, T cell infiltration, NLRP3, cGAS‐STING, oxLDL

## Abstract

The anti‐tumor immune response relies on interactions among tumor cells and immune cells. However, the molecular mechanisms by which tumor cells regulate DCs as well as DCs regulate T cells remain enigmatic. Here, the authors identify a super signaling complex in DCs that mediates the Arf1‐ablation‐induced anti‐tumor immunity. They find that the Arf1‐ablated tumor cells release OxLDL, HMGB1, and genomic DNA, which together bound to a coreceptor complex of CD36/TLR2/TLR6 on DC surface. The complex then is internalized into the Rab7‐marked endosome in DCs, and further joined by components of the NF‐κB, NLRP3 inflammasome and cGAS‐STING triple pathways to form a super signal complex for producing different cytokines, which together promote CD8+ T cell tumor infiltration, cross‐priming and stemness. Blockage of the HMGB1‐gDNA complex or reducing expression in each member of the coreceptors or the cGAS/STING pathway prevents production of the cytokines. Moreover, depletion of the type I IFNs and IL‐1β cytokines abrogate tumor regression in mice bearing the Arf1‐ablated tumor cells. These findings reveal a new molecular mechanism by which dying tumor cells releasing several factors to activate the triple pathways in DC for producing multiple cytokines to simultaneously promote DC activation, T cell infiltration, cross‐priming and stemness.

## Graphical abstract

This study reveals that the Arf1‐ablated tumor cells induce anti‐tumor immune response through releasing oxLDL, HMGB1, and genomic DNA that form a super signal complex and activate the NF‐κB, NLRP3 inflammasome and cGAS‐STING triple pathways in DC for producing CCL5, IL‐1β, type I IFNs, and CXCL10 to simultaneously promote DC activation, T cell infiltration, cross‐priming and stemness.

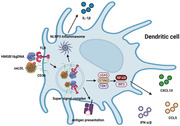

## 1 Introduction

The anti‐tumor immunity is attributed to a sequence of events. The sequence is initiated by type I conventional dendritic cells (cDC1s) to capture, process the cancer‐associated antigens (CAAs) from dying cancer cells, and then present the antigens to naïve T cells within tumor‐draining lymph nodes and prime cancer‐specific CD8^+^ T cells. The cancer antigen‐specifically activated CD8^+^ T cells mobilize and infiltrate into tumors and eliminate cancer cells that display the cognate peptide antigen presented by MHC class I molecules (pMHC). The additional CAAs released by initial dying tumor cells trigger a new round of the sequence and amplify the magnitude of the immune response with each subsequent round.^[^ ^1^ ^]^ However, if tumors persist, CD8^+^ T cells become exhausted, characterized by an orderly loss of effector functions, impaired proliferation, and the upregulation of inhibitory receptors (e.g., PD‐1, Lag‐3, Tim‐3;^[^ ^2^, ^3^ ^]^). But, in both cancer patients and experimental animal models, the tumor‐infiltrating CD8^+^ T cells showed significant heterogeneity. Immunotherapy with immune‐checkpoint blockades (ICBs) and adoptive T cell therapy (ACT) was able to prevent T cell exhaustion and promote complete regression in the therapeutic animal models and certain human tumors.^[^ ^4^, ^5^, ^6^, ^7^, ^8^, ^9^ ^]^

It was found that the stem‐like CD8^+^ T cells mediate responses of ICB and ACT immunotherapies. ICBs both directly promote a proliferative burst in a population of PD‐1^+^ CD8^+^ tumor‐infiltrating stem‐like T cells ^[^ ^5^, ^8^, ^10^, ^11^ ^]^ and also act on metabolic competition and different immune cell types within the tumor microenvironment to promote anti‐tumor effects of the CD8^+^ T cells.^[^ ^12^, ^13^ ^]^ The stem‐like T cells then generate fresh effective T cells to replenish the intratumoral exhausted T cells.^[^ ^5^, ^8^, ^13^, ^14^, ^15^, ^16^, ^17^ ^]^ In lung adenocarcinoma, the migratory conventional type I dendritic cells (cDC1s) enter the tumor‐draining lymph node (dLN) and maintain a reservoir of the stem‐like SlamF6^+^TCF‐1^+^ CD8^+^ T cell population. The stem‐like T cells provide more functional T cell trafficking into the tumor and reduce tumor burden.^[^ ^18^ ^]^

DCs, particularly the conventional type I dendritic cells (cDC1), contribute to anti‐tumor immunity by their ability to present tumor antigens and to secrete cytokines that regulate T cell survival and effector function.^[^ ^19^, ^20^ ^]^ The intra‐tumoral cDC1 attracts T cells into tumors,^[^ ^21^ ^]^ re‐stimulates and expands the tumor‐specific CD8^+^ T cells,^[^ ^22^ ^]^ and supports T cell effector function by promoting T cell stemness.^[^ ^18^ ^]^ DCs are regulated by factors in the tumor microenvironment (TME;^[^ ^23^ ^]^). Tumor cells, including cancer stem cells, often escape from the immune system by secreting immune inhibitory factors and creating an immunosuppressive TME to prevent recruitment and activation of DCs at the tumor site.^[^ ^24^, ^25^, ^26^ ^]^ The development of strategies to increase cDC1 abundance in tumors or facilitate their activation will be key to boosting anti‐tumor immunity and increasing the responsiveness of cancer patients to immunotherapy. The anti‐tumor immune response relies on interactions among tumor cells, DCs, and lymphocytes.

We recently found that the Arf1‐mediated lipid metabolism selectively sustains cancer cells (particularly stem cells) and knockdown of the pathway not only kills cancer cells but also elicits a tumor‐specific immune response that converts dying cancer cells into a therapeutic vaccine, which both significantly increases tumor‐infiltrating and activation of DCs and CD8^+^ T cells.^[^ ^27^, ^28^ ^]^ However, the relationship among tumor cells, DCs, and T cells in activating anti‐tumor immunity in the Arf1‐ablated tumor microenvironment remains unknown. In this study, we found that the immunogenic factors released from the Arf1‐ablated tumor cells induced and activated a super signaling complex in DCs that further promoted T‐cell infiltration, cross‐priming, and stemness through releasing cytokines and chemokines. We found that the Arf1‐ablated tumor cells released HMGB1, oxidized low‐density lipoprotein (oxLDL), and genomic DNA, which might together bind to co‐receptors complex of the CD36/TLR2/TLR6 on the dendritic cell surface. The ligand‐coreceptor super complex then was internalized into the Rab7‐marked endosomes in DCs, and further joined by components of the NF‐κB, NLRP3 inflammasome and cGAS‐STING triple pathways to form a super signal complex for producing CCL5, IL‐1β, type I IFNs and CXCL10, as well as other cytokines through activating the tripe pathways. These inducible cytokines and chemokines promoted CD8^+^ T cell tumor‐infiltrating, tumor antigen‐specific cross‐priming, and stemness for anti‐tumor immunity.

## 2 Results

### 2.1 The Released Factors from the Arf1‐ablated Tumor Cells Activate the NLRP3 Inflammasome, cGAS‐STING, and NF‐kB Triple Pathways in DC

**Figure 1:**
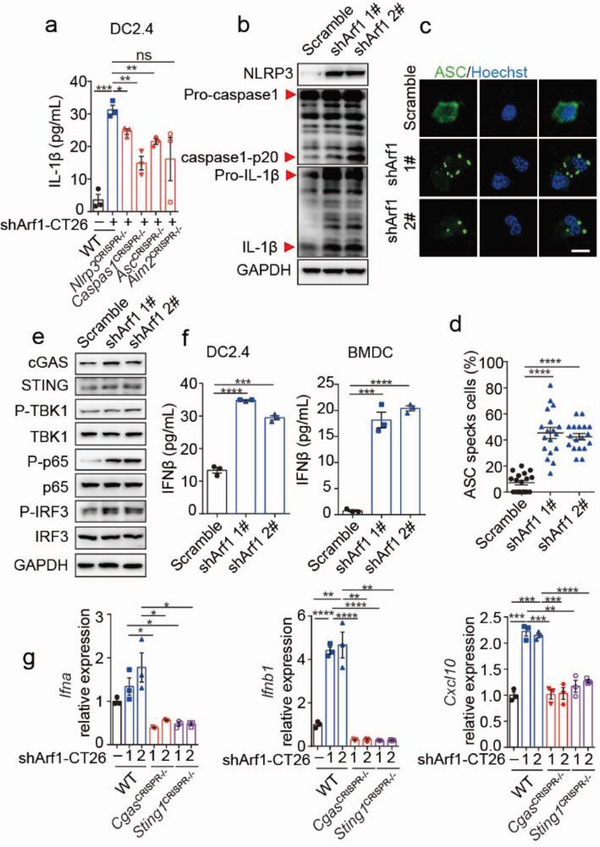
Arf1‐ablated tumor cells activate the NLRP3 inflammasome, cGAS‐STING, and NF‐kB pathways in DCs. a) The secreted IL‐1β was measured from DC2.4 cells that were transfected with control or the indicated constructs and then cocultured with or without Arf1 knockdown CT26 cells for another 24 h (*n =* 3 biological replicates). b) Immunoblotting analysis of NLRP3, pro‐caspase‐1, caspase1‐p20, pro‐IL‐1β and bioactive IL‐1β in DC2.4 cells that were co‐cultured with either the Scramble‐ or Arf1‐ablated CT26 cells. GAPDH was used as a loading control. c, d) Immunofluorescence staining of ASC (c) and quantification of ASC specks (d) in DC2.4 cells after co‐culture with either the Scramble‐ or Arf1‐ablated CT26 cells (*n =* 3). Scale bars, 25 µm. e) Immunoblotting analysis of the indicated proteins in DC2.4 cells after co‐culture with either the Scramble‐ or Arf1‐ablated CT26 cells. f) The secreted IFNβ was measured from DC2.4 or bone marrow‐derived dendritic cells (BMDCs) that were co‐cultured with either the Scramble‐ or Arf1‐ablated CT26 cells (*n =* 3). g) Quantitative Real‐time PCR analysis of the expression of *Ifna*, *Ifnb* and *Cxcl10* in DC2.4 cells that were transfected with or without indicated constructs and then cocultured with either the Scramble‐ or Arf1‐ablated CT26 cells (*n =* 3). Each point represents the percentage of ASC specks per macroscopic field (d). In all of the panels, data are presented as means ± SEMs; *p < 0.05, **p < 0.01, ***p <0.001, ****p < 0.0001. n.s., not significant. Student's *t*‐test.

To investigate the mechanism of anti‐tumor immunity induced by Arf1‐ablation in cancer (stem) cells, we adopted short hairpin RNAs to knock down Arf1 in CT26 colorectal cancer cells and co‐cultured them with DC2.4 dendritic cells (Figure S1a,b, Supporting Information). Using the quantitative real‐time PCR, we examined the expression of genes that encode the inflammatory cytokines and chemokines. Among the tested genes, the expression of Il‐1 family *Il1b* and *Il18*, but not *Tnf* or *Il6*, were significantly increased in DCs that were cocultured with the Arf1‐ablated tumor cells (referred to as Arf1‐ablation‐stimulated DC) in comparison with those in DCs that were cocultured with the Scramble‐ablated control tumor cells (refer to as control DC; Figure S1c,d, Supporting Information). The release of mature IL‐1β and IL‐18 is usually mediated by inflammasomes.^[^ ^29^, ^30^ ^]^ The elevated transcription levels of *Il1b* and *Il18* indicated that the inflammasomes might be activated in the Arf1‐ablation‐stimulated DCs. To confirm this, we compared the protein level of IL‐1β in the Arf1‐ablation‐stimulated and control DCs and found that the protein level of IL‐1β was significantly higher in the Arf1‐ablation‐stimulated DCs than in the control DCs (**Figure** **1**a). Whereas this effect was mostly dependent on NLRP3, not Aim2, inflammasome, since knockout of *Nlrp3*, *Asc*, or *caspase‐1* but not *Aim2* through CRISPR‐Cas9 technique in DC2.4 cells markedly decreased the protein level of IL‐1β induced by the Arf1‐ablated tumor cells (Figure 1a and Figure S1e,f, Supporting Information). In line with this, the Arf1‐ablation‐stimulated DCs had higher levels of cleavage caspase‐1 and bioactive IL‐1β as well as elevated protein levels of NLRP3 than those of the control DCs (Figure 1b). Moreover, we observed more apoptosis‐associated speck‐like protein containing a caspase recruitment domain (ASC) specks in the Arf1‐ablation‐stimulated DCs than those in the control DCs (Figure 1c,d), indicating more effective activation of ASC by the Arf1‐ablated tumor cells. In the previous study, we demonstrated that selective deletion of Arf1 in cancer stem cells induces anti‐tumor immunity and an inflammasome‐mediated cancer cell necrosis/pyroptosis,^[^ ^27^ ^]^ we analyzed cell death in the co‐culture model of Arf1‐ablated tumor cells and DCs (Figure S1b, Supporting Information) and found no significant difference in dying cells between the Arf1‐ablated and control tumor cells (Figure S2a,b, Supporting Information), suggesting that the cancer cell necrosis depends on T cells rather than DCs. Taken together, these data clearly demonstrated that the Arf1‐ablated tumor cells induced NLRP3 inflammasome activation in the co‐cultured DCs.

Interestingly, we also found that the Arf1‐ablated tumor cells induced a significant increase in the expression of *Cxcl10* in the co‐cultured DCs (Figure S1d, Supporting Information). The *Cxcl10* promoter contains interferon regulatory factor (IRF) binding sites,^[^ ^31^ ^]^ indicating that IRF transcription factors may regulate *Cxcl10* expression. Moreover, the chemokine CXCL10 was considered as a marker of cyclic GMP‐AMP synthase (cGAS) activation.^[^ ^32^ ^]^ The activation of cGAS signaling always leads to downstream stimulator of interferon genes (STING) activation, which then activates IRFs.^[^ ^33^ ^]^ Thus, the cGAS‐STING signaling pathway might regulate *Cxcl10* expression through IRFs in the Arf1‐ablation‐stimulated DCs. To test this possibility, we examined the protein levels of this signaling cascade in the Arf1‐ablation‐stimulated DCs. We observed a significant increase in the protein levels of cGAS and STING, as well as phosphorylation of the TANK‐binding kinase 1 (TBK1) and IRF3 (Figure 1e), indicating that the cGAS‐STING signaling pathway was activated in the Arf1‐ablation‐stimulated DCs. Moreover, in comparison with the control DCs, both DC2.4 dendritic cells and bone marrow‐derived dendritic cells (BMDCs) significantly increased IFNβ secretion after co‐culture with the Arf1‐ablated tumor cells (Figure 1f). Co‐culturing with the Arf1‐ablated tumor cells led to significantly increased expression of *Ifna*, *Ifnb*, and *Cxcl10* in the DCs (Figure 1g), while this effect was abolished after knocking down *Cgas* or *Sting1* genes in the DCs (Figure 1g and Figure S4a, Supporting Information), suggesting that the co‐cultured DCs produced type I IFNs and *Cxcl10* in a cGAS‐STING signaling dependent manner. To further confirm that the Arf1‐ablated tumor cells induced activation of the cGAS‐STING signaling pathway in DCs, we sorted DCs from tumors of mice implanted with either Scramble‐ablated or Arf1‐ablated tumor cells and examined the expression of target genes regulated by the cGAS‐STING pathway by RT‐qPCR. We found that the Arf1‐ablation‐stimulated DCs had higher expression of *Ifna* and *Cxcl10* as well as genes induced by type I IFNs (*Ifit1* and *RASD2)* than those in the control DCs (Figure S3, Supporting Information). Together, these results demonstrated that the Arf1‐ablated tumor cells induced the activation of the cGAS‐STING signaling pathway in DCs both in vitro and in vivo.

*Ccl5* is known as a target gene of the transcription factor NF‐κB. Therefore, the increased expression of *Ccl5* in the Arf1‐ablation‐stimulated DCs (Figure S1d, Supporting Information) might be due to activation of the NF‐κB pathway, as evidenced by elevated levels of the phosphorylated p65 (Figure 1e). Altogether, we concluded that the Arf1‐ablated tumor cells were able to activate the NLRP3 inflammasome, cGAS‐STING, and NF‐κB pathways in DCs, the triple pathways together induced production of the cytokines and chemokines in the activated DCs to activate tumor immune microenvironment in the Arf1‐deficient‐tumors.

Our above data demonstrated that the Arf1‐ablated tumor cells induced activation of the NF‐κB, NLRP3 inflammasome, and cGAS‐STING triple pathways in DCs. We further investigated the relationship among the triple pathways. We found that either knockdown of *Cgas* or *Sting1* in DCs or pharmacological inhibition of the cGAS‐STING signal pathway could significantly prevent NLRP3 inflammasome activation in the Arf1‐ablation‐stimulated DCs (Figure S4a–d, Supporting Information). However, CRISPR‐Cas9 mediated knockout of *Nlrp3*, *Asc*, or *caspase‐1* failed to prevent IFNβ secretion from the Arf1‐ablation‐stimulated DCs (Figure S4e, Supporting Information). These results indicated that the cGAS‐STING pathway might function upstream of the NLRP3 inflammasome. Furthermore, we found that the blocking function of type I IFNs by anti‐IFNAR1 antibodies abrogated the elevation of phosphorylated STAT2, NLRP3, pro‐caspase‐1, and IL‐1β in the Arf1‐ablation‐stimulated DCs (Figure S4f, Supporting Information). It was previously reported that the NF‐κB signaling could prime the NLRP3 inflammasomes by inducing the expression of NLRP3, pro‐IL‐1β , and pro‐IL‐18^[^ ^34^ ^]^. These data together suggested that the cGAS‐STING and NF‐κB pathways might function upstream and prime the NLRP3 inflammasome in DCs when it was activated by the Arf1‐ablated tumor cells.

### 2.2 The Tumor‐Released HMGB1‐gDNA Complex and OxLDL Activate the Triple Pathways in DCs

**Figure 2:**
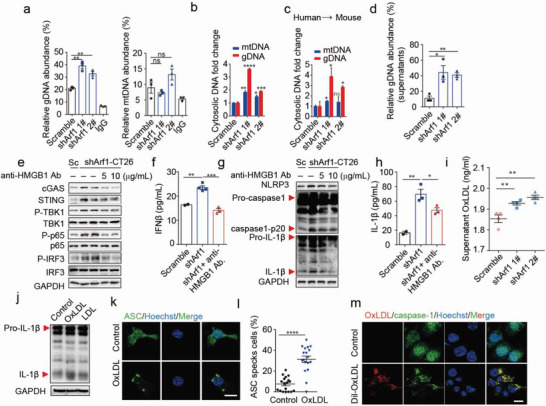
The tumor‐released HMGB1‐gDNA complex activates cGAS‐STING and NF‐kB pathways, and the tumor‐released oxLDL induces inflammasome activation in DCs. a) Relative abundance of gDNA and mtDNA in the cGAS immunoprecipitates of DC2.4 cells that were cocultured with either the Scramble‐ or Arf1‐ablated CT26 cells (*n =* 3). b) Relative abundance of cytosolic gDNA and mtDNA in DC2.4 cells that were cocultured with either the Scramble‐ or Arf1‐ablated CT26 cells (*n =* 3). c) DC2.4 cells were cocultured with either the Scramble‐ or Arf1‐ablated human DLD1 colorectal tumor cells, the cytosolic human gDNA, and mtDNA in DCs were examined by RT‐qPCR (*n =* 3). d) Relative abundance of gDNA in the HMGB1 immunoprecipitates of the collected culture medium from either the Scramble‐ or Arf1‐ablated CT26 cells (*n =* 3). e) Immunoblotting analysis of the indicated proteins in DC2.4 cells that were cocultured with either the Scramble (Sc)‐ or Arf1‐ablated CT26 cells in presence or absence of the indicated concentrations of anti‐HMGB1 antibody. f) The secreted IFNβ was measured from DC2.4 cells that were treated as in (e) (*n =* 2‐4). g) Immunoblotting analysis of the indicated proteins in DC2.4 cells that were treated as in (e). h) The secreted IL‐1β was measured from DC2.4 cells that were treated as in (e) (*n =* 2‐3). i) The secreted oxLDL was measured from the medium of either the Scramble‐ or Arf1‐ablated CT26 cells (*n =* 4). j) Immunoblotting analysis of pro‐IL‐1β and bioactive IL‐1β in DC 2.4 cells that were treated with or without oxLDL and LDL. k, l) Immunofluorescence staining of ASC (k) and quantification of ASC specks (l) in DC2.4 cells that were treated with or without exogenous oxLDL. Scale bars, 25 µm. m) Immunofluorescence staining of caspase‐1 and oxLDL in DC2.4 cells that were treated with or without exogenous Dil‐OxLDL. Scale bars, 25 µm. Each point represents the percentage of ASC specks per macroscopic field (l). In all of the panels, data are presented as means ± SEMs; *p < 0.05, **p < 0.01, ***p <0.001, ****p < 0.0001. n.s., not significant. Student's *t*‐test.

Next, we sought to identify factors released from the Arf1‐ablated tumor cells that activated the triple pathways in DCs. cGAS acts as a cytosolic DNA sensor, both genomic DNA (gDNA) and mitochondrial DNA (mtDNA) bind to and activate cGAS to initiate downstream signaling.^[^ ^35^ ^]^ To determine whether the tumor cell‐derived DNAs were involved in cGAS activation or not, we performed a pulldown assay to assess the DNA‐protein interaction in either the Arf1‐ablation‐stimulated or control DCs. We examined the amount of gDNA or mtDNA in the cGAS complex and found that gDNA but not mtDNA was enriched in the cGAS immunoprecipitates in the Arf1‐ablation‐stimulated DCs in comparison with that in the control DCs (**Figure** **2**a). To confirm whether the tumor cell‐derived DNAs truly entered into the co‐cultured DCs or not, we extracted DNAs from the cytoplasm of either the Arf1‐ablation‐stimulated or control DCs. We found that both gDNAs and mtDNAs were significantly increased in the cytoplasm of the Arf1‐ablation‐stimulated DCs in comparison with those in the control DCs (Figure 2b), with gDNAs displaying more increase (Figure 2b). In order to directly prove that the cytoplasm DNAs in DCs came from the Arf1‐ablated tumor cells, we co‐cultured mouse DCs with human DLD1 colorectal cancer cells that were transfected with either shScramble or shArf1 (Figure S1a, Supporting Information). We found that the human gDNAs were significantly increased in the cytoplasm of mouse DCs that were co‐cultured with the Arf1‐ablated human DLD1 cancer cells in comparison with those in the cytoplasm of mouse DCs that were co‐cultured with the Scramble‐ablated human DLD1 tumor cells (Figure 2c). These data together demonstrated that the tumor‐derived gDNAs, but not mtDNAs, activated cGAS in the Arf1‐ablation‐stimulated DCs.

A recent study demonstrated that the nucleic acid‐peptide complex had higher stability and improved antigenicity to efficiently initiate innate immune responses than the nucleic acid alone.^[^ ^36^ ^]^ Interestingly, we found that inhibition or knockdown of Arf1 in CT26 cells led to HMGB1 translocation from nucleus to the cytoplasm (Figure S5a–c, Supporting Information). Meanwhile, the amount of HMGB1 in the culture medium of the Arf1‐ablated tumor cells was significantly increased (Figure S5d, Supporting Information). HMGB1 binds preferentially to a non‐double helix form of DNA.^[^ ^37^ ^]^ This prompted us to further examine whether   the HMGB1‐gDNA complex was originally released by the Arf1‐ablated tumor cells or not. By chromatin immunoprecipitation and RT‐qPCR analysis, we found a significant increase of gDNA in the HMGB1 immunoprecipitates from medium of the Arf1‐ablated tumor cells in comparison with that of the control cells (Figure 2d), indicating that the HMGB1‐gDNA complex was released into extracellular space from the Arf1‐ablated tumor cells.

To examine whether the HMGB1‐gDNA complex was responsible for activation of the cGAS‐STING‐type I IFN signaling pathway in the Arf1‐ablation‐stimulated DCs or not. We cultured either the control or Arf1‐ablation‐stimulated DCs in the presence or absence of anti‐HMGB1 antibodies. We found that the addition of the anti‐HMGB1antibody significantly blocked the elevation of cGAS and STING proteins, as well as the increased phosphorylation of TBK1 and IRF3 in the Arf1‐ablation‐stimulated DCs (Figure 2e). Blockage of the HMGB1‐gDNA complex by the anti‐HMGB1 antibodies also prevented production of type I IFNs and *Cxcl10* in the Arf1‐ablation‐stimulated DCs (Figure 2F and Figure S6a, Supporting Information). These results together demonstrated that the HMGB1‐gDNA complex released from the Arf1‐ablated tumor cells activated the cGAS‐STING‐ type I IFNs in DCs.

Interestingly, the depletion of HMGB1 also significantly abrogated an increase in the protein levels of caspase‐1 p20, active IL‐1β, and NLRP3 in the Arf1‐ablation‐stimulated DCs (Figure 2g,h). In addition, the anti‐HMGB1 antibody treatment also blocked elevation of the mRNA levels of *Il1b*, and *Il18* in the Arf1‐ablation‐stimulated DCs (Figure S6b, Supporting Information). Since NLRP3 could also be activated by oxidative DNA,^[^ ^31^ ^]^ we further examined the tumor‐derived oxidative DNA by using a specific antibody in the Arf1‐ablation‐stimulated DCs. However, we only observed minimal overlap between NLRP3 and the oxidative tumor‐DNA in the Arf1‐ablation‐stimulated DCs (Figure S7, Supporting Information). Therefore, inflammasome inactivation after HMGB1 depletion might be due to reduced priming of NLRP3 inflammasome by the HMGB1‐activated cGAS‐STING and NF‐kB pathways (Figure S4, Supporting Information).

Next, we sought to identify the tumor‐derived factors that engaged in direct activation of the NLRP3 inflammasome in the co‐cultured DCs. A recent study reported that endogenous damage‐associated molecular patterns (DAMPs), such as reactive oxygen species (ROS) and oxidized lipids, could activate inflammasome in the tumor‐infiltrating DCs.^[^ ^38^ ^]^ Arf1 had a critical role in mediating lipolysis and its ablation resulted in ROS increase and lipid droplet accumulation.^[^ ^27^, ^28^ ^]^ The elevated ROS may further promote lipid oxidation. Therefore, the oxidized lipids might be involved in activation of the inflammasome in the Arf1‐ablation‐stimulated DCs. To investigate this possibility, we first examined oxidized lipids in the Arf1‐ablated CT26 tumor cells and observed a significant increase in the BD‐C11 ratio (green to red) in the Arf1‐ablated tumor cells in comparison with that in the control cells (Figure S8a,b, Supporting Information), indicating increased lipid peroxidation. In line with this, the concentration of oxidized low‐density lipoprotein (oxLDL), which contains oxidized lipids, was markedly increased in the supernatants of the Arf1‐ablated tumor cells in comparison with that in the control cells (Figure 2i). In order to determine whether oxLDL directly activated the inflammasome or not, we incubated DC2.4 cells with or without exogenous oxLDL and found that oxLDL alone, but not LDL, was sufficient to induce inflammasome activation in DCs, as evidenced by increased production of active IL‐1β (Figure 2j), as well as increased ASC specks (Figure 2k,l). Moreover, we observed an overlap of exogenous oxLDL with caspase‐1 in the DCs (Figure 2m), suggesting that oxLDL might directly bind to caspase‐1 and induce assembly and activation of the inflammasome in DCs.^[^ ^29^ ^]^

Notably, DCs treated with oxLDL alone did not induce an increase in the expression of *Ccl5*, *Ifna*, *Ifnb*, *and Cxcl10* compared to the untreated cells (Figure S8c, Supporting Information), indicating thatoxLDL did not contribute to the activation of the cGAS‐STING‐type I IFNs or NF‐κB signaling pathways in DCs. A previous study reported that HMGB1 could bind Toll‐like receptors (TLRs) and induce activation of the TLR‐MyD88‐NF‐κB signaling pathway for producing inflammatory cytokines such as *Ccl5* ^[^ ^39^ ^]^. In line with those findings, we observed that blockage of HMGB1 alone was sufficient to prevent p65 phosphorylation (Figure 2e) and abrogated the production of *Ccl5* (Figure 2e and Figure S6b, Supporting Information) in the Arf1‐ablation‐stimulated DCs. Taken together, these data demonstrated that the tumor‐derived HMGB1‐gDNA complex engaged in activation of the cGAS‐STING‐type I IFNs and NF‐κB pathways that further primed the inflammasome, which was finally activated by the tumor‐derived oxLDL in DCs.

### 2.3 The Coreceptors of CD36/TLR2/TLR6 Mediate Tumor Cell to DC Signal from the Arf1‐Ablated Tumor Cells

**Figure 3:**
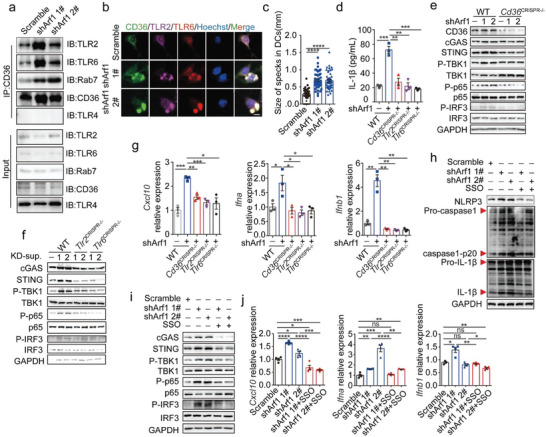
A co‐receptor complex of CD36/TLR2/TLR6 mediates the tumor‐derived signals and activates the triple pathways in DC. a) Co‐immunoprecipitation experiments to demonstrate binding of CD36 to TLR2, TLR6, TLR4 and Rab7 in DC2.4 cells that were co‐cultured with either the Scramble‐ or Arf1‐ablated CT26 cells. b, c) Immunofluorescence staining of CD36, TLR2 and TLR6 (b) and quantification of CD36/TLR2/ TLR6 specks (c) in DC2.4 cells that were treated as in (a) (*n =* 3). Scale bars, 25 µm. d) The secreted IL‐1β level was measured from DC2.4 cells that were transfected with or without the indicated constructs and then cocultured with either the Scramble‐ or Arf1‐ablated CT26 cells (*n =* 3). e, f) Immunoblotting analysis of the indicated proteins in DC2.4 cells that were treated as in (d). 1 indicates shArf1 #1‐ablated CT26 cells and 2 indicates shArf1 2#‐ablated CT26 cells. g) RT‐qPCR analysis of the expression of *Cxcl10*, *Ifna* and *Ifnb* in DC2.4 cells that were treated as in (d) (*n =* 3). h, i) Immunoblotting analysis of the indicated proteins in DC2.4 cells that were cocultured with either the Scramble‐ or Arf1‐ablated CT26 cells in the presence or absence of CD36‐specific inhibitor SSO (20 µM). j) RT‐qPCR analysis of the expression of *Cxcl10*, *Ifna* and *Ifnb* in DC2.4 cells that were treated as in (h) (*n =* 3). Each point represents the size of CD36/TLR2/TLR6 specks (c). In all of the panels, data are presented as means ± SEMs; *p < 0.05, **p < 0.01, ***p <0.001, ****p < 0.0001. n.s., not significant. Student's *t*‐test.

Next, we sought to identify the receptors that might mediate signals from tumor cells to DC for activation of the Arf1‐ablation‐stimulated DCs. The scavenger receptor CD36 is a transporter of free fatty acids and oxidized lipids such as oxLDL.^[^ ^40^ ^]^ We found that the Arf1‐ablation‐stimulated DCs exhibited markedly increased expression of *Cd36* in comparison with that of the control DCs (Figure S9a, Supporting Information). Moreover, incubation of DC2.4 cells withoxLDL alone also strongly elevated the expression of *Cd36* (Figure S9b, Supporting Information). These results suggested that the CD36 receptor might engage in activation of the Arf1‐ablation‐stimulated DCs. It was previously reported that CD36 cooperated with a TLR4/TLR6 heterodimer to mediate inflammatory cytokines secretion by macrophages under the stimulation of oxidized lipids.^[^ ^41^ ^]^ TLRs such as TLR2 and TLR4 were also demonstrated as the receptors of HMGB1^[^ ^39^ ^]^. We therefore detected the expression of TLRs in DCs. Interestingly, we found that the expression of *Tlr2* and *Tlr6* was significantly increased in the Arf1‐ablation‐stimulated DCs in comparison with that in the control DCs (Figure S9c, Supporting Information), while the expression of *Tlr4* was moderately increased, and the expression of *Tlr9* was decreased (Figure S9c, Supporting Information). Moreover, the over‐expressed TLR2 colocalized with the tumor‐derived DNA in DCs (Figure S10a, Supporting Information). To determine whether CD36 formed a coreceptor complex with members of the TLR family or not in the Arf1‐ablation‐stimulated DCs, we performed co‐immunoprecipitation assays. We found increased binding of CD36 to TLR2 and TLR6, but not to TLR4, in the Arf1‐ablation‐stimulated DCs (**Figure** **3**a and Figure S9d,e, Supporting Information). By immunofluorescence, we observed that CD36 colocalized with TLR2 and TLR6 in the Arf1‐ablation‐stimulated DCs (Figure 3b). These findings suggest that CD36 formed a receptors complex with TLR2 and TLR6 in the Arf1‐ablation‐stimulated DCs. Moreover, a significant increase in the size of specks of the CD36/TLR2/TLR6 complex was observed in the Arf1‐ablation‐stimulated DCs in comparison with that of the control DCs (Figure 3c), indicating that the receptor complex might mediate the endocytosis process of DCs.

To investigate whether the receptors complex of CD36/TLR2/TLR6 was responsible for activation of the Arf1‐ablation‐stimulated DCs. We first knocked out *Cd36*, *Tlr2*, or *Tlr6* in DCs by CRISPR‐Cas9 technique and found that the ASC speck formation was significantly reduced in the *Cd36* ^CRISPR‐/−^, *Tlr2* ^CRISPR‐/−^ or *Tlr6* ^CRISPR‐/−^ DCs in comparison with that in wild‐type DCs after co‐culture with the Arf1‐ablated tumor cells (Figure S10b–d, Supporting Information). Moreover, the deficiency of *Cd36*, *Tlr2*, or *Tlr6* abolished IL‐1β secretion from the Arf1‐ablation‐stimulated DCs (Figure 3d). Consistent with these results, we observed that the deletion of *Cd36*, *Tlr2*, or *Tlr6* significantly reduced the increased protein levels of cGAS, STING, the increased phosphorylation of TBK1, p65, and IRF3, as well as the increased transcriptional levels of type I IFNs and *Cxcl10* in the Arf1‐ablation‐stimulated DCs (Figure 3e‐g). Collectively, these data demonstrated that the coreceptors of CD36/TLR2/TLR6 were responsible for the activation of the inflammasome, cGAS‐STING, and NF‐κB pathways in the Arf1‐ablation‐stimulated DCs. To further confirm this, we pharmacologically inhibited the functions of the coreceptors in the co‐culture systems. We found that treatment of DC2.4 cells with the CD36 irreversible inhibitor sulfosuccinimidyl oleate (SSO) significantly reduced the increased protein levels of NLRP3, caspase‐1 p20 and active IL‐1β (Figure 3h), cGAS and STING, phosphorylation of TBK1, p65 and IRF3 (Figure 3i), and the increased production of Il‐1 family cytokines, type I IFNs, *Cxcl10* and *Ccl5* (Figure  3j and Figure S10e, Supporting Information), in the Arf1‐ablation‐stimulated DCs. Similar to CD36 inhibitor SSO, TLR2/6 specific inhibitors C29 or MMG‐11 treatment also markedly blocked elevation of the protein levels of NLRP3 inflammasome and cGAS‐STING pathway cascades (Figure S10f,h, Supporting Information), as well as the increased production of Il‐1 family cytokines, type I IFNs, *Cxcl10* and *Ccl5* (Figure S10g,i, Supporting Information), in the Arf1‐ablation‐stimulated DCs. Taken together, these data demonstrated that the inducible coreceptors of CD36/TLR2/TLR6 were responsible for the activation of the Arf1‐ablation‐stimulated DCs.

### 2.4 The Ligand‐Coreceptor Complex was Internalized and Sensed by Cytosolic Sensors in DC Endosomes

**Figure 4:**
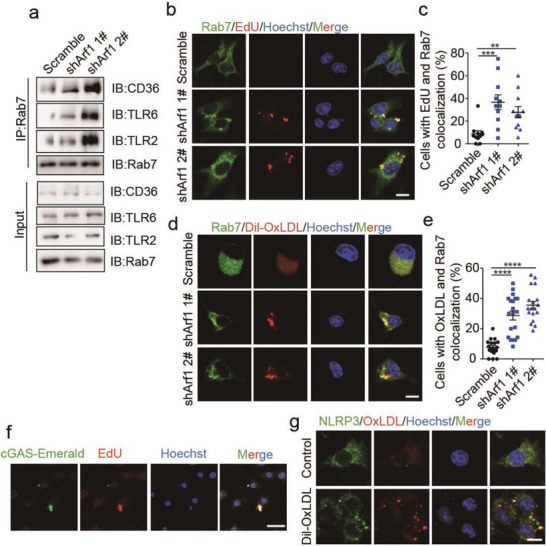
The ligand‐coreceptor complex was recruited into the late endosome and formed a super signal complex. a) Co‐immunoprecipitation experiments to demonstrate binding of Rab7 to TLR2, TLR6 and CD36 in DC2.4 cells that were cocultured with either the Scramble‐ or Arf1‐ablated CT26 cells. b, c) Immunofluorescence staining of Rab7 and EdU (b) and quantification of colocalized cells (c) in DC2.4 cells that were coculture with either the Scramble‐ or Arf1‐ablated CT26 cells (*n =* 3). Scale bars, 25 µm. d, e) Immunofluorescence staining of Rab7 and oxLDL (b) and quantification of colocalized cells (c) in DC2.4 cells that were cocultured with either the Scramble‐ or Arf1‐ablated CT26 cells in the presence of 20 µM Dil‐oxLDL (*n =* 3). Scale bars, 25 µm. f) Immunofluorescence staining of EdU and cGAS in DC2.4 cells. DC2.4 dendritic cells were transfected with the cGAS‐Emerald constructs and then cocultured with Arf1‐ablated CT26 cells in the presence of 10 µM EdU for 24 h. Scale bars, 25 µm. g) Immunofluorescence staining of NLRP3 and oxLDL in DC2.4 cells that were treated with or without Dil‐oxLDL for 24 h. Scale bars, 25 µm. Each point represents the percentage of colocalized cells in DC2.4 cells (c, e). In all of the panels, data are presented as means ± SEMs; **p < 0.01, ***p <0.001, ****p < 0.0001. Student's *t*‐test.

Next, we investigated the molecular mechanisms of DC activation by the tumor‐derived ligands and the DC‐produced inducible coreceptors. A previous study reported that macrophages engulfed tumor cell(s) and recruited the tumor cargoes into phagosomes.^[^ ^42^ ^]^ In line with this, we observed that the coreceptor complex formed specks in the Arf1‐ablation‐stimulated DCs (Figure 3b,c). This prompted us to examine whether the ligand‐coreceptors complex was recruited into phagosomes or not in DCs. We analyzed the immunoprecipitates of CD36, TLR2, TLR6, and Rab7 (a marker of phagosome/endosome) by western blotting. Interestingly, we found that Rab7 is directly bound to each member of the coreceptors (Figure 3a and Figure S9d,e, Supporting Information). The interaction between Rab7 and the coreceptors was further confirmed in the Rab7‐immunoprecipitates of the Arf1‐ablation‐stimulated DCs (**Figure** **4**a), indicating that the coreceptors were recruited into the phagosomes in the Arf1‐ablation‐stimulated DCs. Consistently, we observed that the ligands of both tumor‐derived DNAs and exogenous oxLDL also colocalized with Rab7 in the DCs (Figure 4b‐e). Moreover, the over‐expressed cGAS and cytosolic sensor NLRP3 colocalized with the tumor‐derived DNA and exogenous oxLDL in DCs, respectively (Figure 4f,g). The disrupted membrane of phagosomes might facilitate the recognition of cargoes and their cytosolic sensors in DCs (Figure S11, Supporting Information). Together, these results suggested such a model, in which the ligand‐coreceptor complex was internalized into DC and then recruited into phagosomes, where free ligands were respectively recognized by different cytosolic sensors to activate the downstream pathways.

### 2.5 The Activated DCs have the Enhanced Capacity to Cross‐Prime Tumor Antigen‐Specific CD8^+^ T Cells

**Figure 5:**
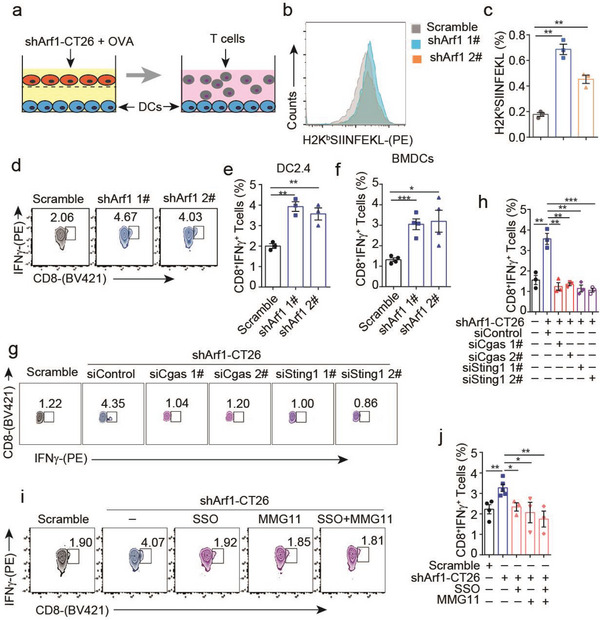
Activated DCs by the Arf1‐ablated tumor secretion had enhanced capacity to cross‐prime the tumor antigen‐specific CD8^+^T cells. a) Experimental design: DC2.4 cells or BMDCs were pre‐cocultured with or without Arf1‐ablated tumor cells in the presence of 100 µM OVA for 24 h and then re‐cocultured with the naïve CD8^+^T cells isolated from the spleens of OT1 mice for another 3 days. b, c) Flow cytometry analysis of H2K^b^SIINFEKL expression (b) and quantification of cells expressing H2K^b^SIINFEKL(c) in the naïve CD8^+^T cells after coculture with DC2.4 cells treated as described in (a) for 3 days (*n =* 3). d‐f) Flow cytometry analysis of IFNγ expression (d) and quantification of cells expressing IFNγ (e, f) in the naïve CD8^+^ OT1 T cells that were cocultured with DC2.4 cells (e) or BMDCs (f) that were treated as described in (a) (*n =* 3). g, h) Flow cytometry analysis of IFNγ expression (g) and quantification of cells expressing IFNγ (h) in the naïve CD8^+^ OT1 T cells after coculture with BMDCs that were transfected with the indicated siRNAs and then treated as described in (a) (*n =* 3). i, j) Flow cytometry analysis of IFNγ expression (i) and quantification of cells expressing IFNγ (j) in the naïve CD8^+^ OT1 T cells after coculture with BMDCs that were treated as described in (a) in the presence or absence of the indicated inhibitors (*n =* 3). In all of the panels, data are presented as means ± SEMs; *p < 0.05, **p < 0.01, ***p <0.001, ****p < 0.0001. Student's *t*‐test.

We further investigated whether the activated DCs have enhanced capacity to cross‐prime CD8^+^ T cells or not. For doing this, the DC2.4 cells were first pre‐cocultured with either the control or Arf1‐ablated tumor cells in the presence of ovalbumin (OVA) and then re‐cocultured with naïve CD8^+^ T cells isolated from the spleens of OT1 mice (**Figure** **5**a). We analyzed the frequency of SIINFEKLMHC‐I tetramer expressing CD8^+^ T cells in total naïve CD8^+^ OT1 T cells and found that the frequency of SIINFEKLMHC‐I tetramer expressing CD8^+^T cells was significantly increased in the naïve CD8^+^ OT1 T cells that were cocultured with the Arf1‐ablation‐stimulated‐DCs (Figure 5b,c), indicating increased tumor antigen‐specific CD8^+^ T cells. Moreover, CD8^+^ OT1 T cells that were co‐cultured with the Arf1‐ablation‐stimulated‐DCs were more active and produced more IFNγ (Figure 5d,e). To confirm this result, we also pre‐cocultured BMDCs with either the control or Arf1‐ablated tumor cells in the presence of OVA and then re‐cocultured the treated BMDCs with naïve CD8^+^ OT1 T cells, we observed similar increases of the tumor antigen‐specific CD8^+^ T cells and IFNγ production (Figure 5f). Notably, these enhanced capabilities of DCs were dependent on the cGAS‐STING signaling pathway, since knockdown of cGAS or STING in BMDCs significantly decreased the frequency of IFNγ^+^ CD8^+^ T cells (Figure 5g,h). Consistent with these results, pharmacological inhibition of the function of CD36, TLR2/6, or the coreceptors of CD36/TLR2/TLR6 abolished the enhanced capacities of BMDCs that were co‐cultured with the Arf1‐ablated tumor cells (Figure 5i,j). These results together demonstrated that the Arf1‐ablation‐stimulated DCs have enhanced capacities of cross‐priming and activating naïve CD8^+^ T cells.

### 2.6 IL‐1β and type I IFNs Released from the Activated DCs are Responsible for Maintenance of the Stem Cell‐like CD8+ T Cells

**Figure 6:**
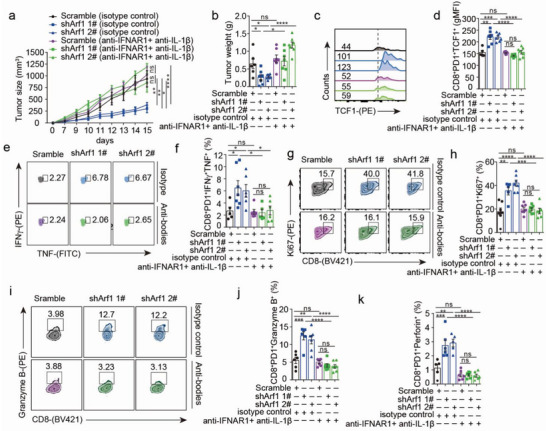
Activated DC‐released cytokines are responsible for maintenance of the anti‐tumor stem cell‐like CD8^+^ T cells. a) BALB/c mice were transplanted with either the Scramble‐ or Arf1‐ablated CT26 cells and treated with 200 µg of anti‐IFNAR1 and anti‐IL‐1β antibodies or isotype control antibody on days 0, 2, and 4. Tumor growth was measured over time (*n =* 6–7 mice). b) Tumor weight was measured from mice that were treated with or without anti‐IFNAR1 and anti‐IL‐1β antibodies (*n =* 6–7 mice). c, d) Flow cytometry analysis of TCF1 expression (c) and quantification of cells expressing TCF1 (d) among the tumor‐infiltrating PD1^+^CD8^+^ T cells (*n =* 6–7 mice). e, f) Flow cytometry analysis of TNF and IFNγ expression (e) and quantification of cells expressing TNF and IFNγ (f) among the tumor‐infiltrating PD1^+^CD8^+^ T cells (*n =* 5–7 mice). g, h) Analysis of Ki67 expression (g) and quantification of cells expressing Ki67 (h) among the tumor‐infiltrating PD1^+^CD8^+^ T cells (*n =* 6‐7 mice). i–k) Flow cytometry analysis of the tumor‐infiltrating CD8^+^ T cells from mice that were transplanted with either the Scramble‐ or Arf1‐ablated CT26 cells and treated with or without anti‐IFNAR1 and anti‐IL‐1β antibodies. Expression of Granzyme B (i, j) and perforin (k) were shown (*n =* 5‐7 mice). In all of the panels, data are presented as means ± SEMs; *p < 0.05, **p < 0.01, ***p <0.001, ****p < 0.0001. n.s., not significant. *P* values were determined by two‐way ANOVA with Tukey's multiple comparisons tests (a), or unpaired two‐tailed Student's *t*‐test (b, d, f, h. j, k).

We next examined whether the activated DCs contributed to the anti‐tumor immunity induced by the Arf1‐ablated tumor cells. Our above‐given data have shown that the Arf1‐ablation‐stimulated DCs produced a great amount of IL‐1β and type I IFNs, both of which have critical roles in mediating anticancer immune responses.^[^ ^43^, ^44^ ^]^ We supposed that the IL‐1β and type I IFNs cytokines might be responsible for anti‐tumor immunity induced by the Arf1‐ablated tumor cells. To test this hypothesis, we subcutaneously inoculated BALB/c mice with the control or Arf1‐ablated CT26 cells, and then administrated mice with anti‐IFNAR1 and anti‐IL‐1β antibodies or isotype control antibodies at day 0, 2, and 4 after tumor cell injection, respectively. In the isotype treatment groups, a remarkable reduction in tumor size and a significant decrease in tumor weight were observed in mice that received the Arf1‐ablated tumor cells in comparison with mice that received the control tumor cells (**Figure** **6**a,b). These results suggested that the Arf1‐ablated tumor cells induced an anti‐tumor immunity in vivo. However, compared to the isotype treatment, treatments with systemic anti‐IFNAR1 and anti‐IL‐1β antibodies abrogated anti‐tumor effects, as evidenced by significantly increased tumor size and tumor weight of mice bearing the Arf1‐ablated tumor cells (Figure 6a,b).

Previous studies reported that type I IFNs promote naïve CD8^+^ T cell differentiation and stem cell‐like T cell maintenance,^[^ ^45^ ^]^ and IL‐1β regulates tumor antigen‐specific T cell polarization and proliferation.^[^ ^46^ ^]^ Moreover, a recent study demonstrated that inflammasome‐mediated IL‐1β could maintain or induce stem cell‐like CD8^+^ T cells in the tumor microenvironment.^[^ ^38^ ^]^ To investigate whether the type I IFNs and IL‐1β cytokines mediate the Arf1‐ablated tumor cell‐induced antitumor immunity via regulating the stem cell‐like CD8^+^ T cells or not, we analyzed the PD‐1 positive CD8^+^ T cells in tumors of mice treated with or without anti‐IFNAR1 and anti‐IL‐1β antibodies. We observed a significant increase in the frequency of PD1^+^CD8^+^ T cells in tumors of mice bearing the Arf1‐ablated tumor cells, whereas the increase was completely abolished after treatment with both anti‐IFNAR1 and anti‐IL‐1β antibodies (Figure S12a, Supporting Information). It was previously reported that the PD1^+^CD8^+^T cells contain both stem cell‐like and effector‐like CD8^+^ T cells.^[^ ^13^ ^]^ Interestingly, we found that all grafts with the Arf1‐ablated CT26 cells had significantly reduced the TCF1^+^PD1^+^CD8^+^T cells after treatment with anti‐IFNAR1 and anti‐IL‐1β antibodies in comparison with the treatment of the isotype control (Figure 6c,d and Figure S12b, Supporting Information). In line with this, treatment of mice bearing the Arf1‐ablated tumor cells with anti‐IFNAR1 and anti‐IL‐1β antibodies had markedly reduced frequency of the TNF^+^IFNg^+^PD1^+^CD8^+^ T cells in tumors in comparison with those in tumors treated with the isotype control antibody (Figure 6e,f). These results indicated that the depletion of type I IFNs and IL‐1β cytokines decreased the stem cell‐like CD8^+^ T cells in tumors of mice bearing the Arf1‐ablated tumor cells. To further address the role of IL‐1β and type I IFNs secreted by DC cells in specifically regulating CD8^+^T cell stemness, we selectively knocked down *Il1b* or *Ifna/b* in dendritic cells to get *Il1b*‐knockdown and *Ifna/b*‐knockdown dendritic cells, respectively. First, we co‐cultured these gene‐knockout dendritic cells with either the Arf1‐ablated tumor cells or scrambled control tumor cells for 24 h to induce dendritic cell activation. Then, the tumor cell‐challenged dendritic cells were re‐cocultured with OT1 T cells for another 5 days, and the stemness markers expressed by T cells were determined by flow cytometry. We found that the Arf1‐ablated tumor cell‐challenged dendritic cells could promote expression of T cell stemness marker TCF1 in comparison with that of scramble tumor cell‐challenged dendritic cells (Figure S12f,g, Supporting Information). Knockdown of *Il1b* or *Ifna/b* in dendritic cells significantly blocked the induction of T cell stemness marker TCF1 by the Arf1‐ablated tumor cell‐challenged dendritic cells (Figure S12f,g, Supporting Information). Together, these data clearly demonstrated that both IL‐1β and type I IFNs released from the activated DCs were responsible for the induction and/or maintenance of the stem cell‐like CD8^+^ T cells.

In tumors of mice bearing the Arf1‐ablated tumor cells, the expression of Ki67 and T‐bet was significantly increased in the PD1^+^CD8^+^T cells, but this was abolished after treatment with anti‐IFNAR1 and anti‐IL1β antibodies (Figure 6g,h and Figure S12c,d, Supporting Information), suggesting that the type I IFNs and IL‐1β cytokines are responsible for maintenance of the effective CD8^+^T cells in mice bearing the Arf1‐ablated tumor cells. Moreover, compared to isotype control antibody treatment, treatment with systemic anti‐IFNAR1 and anti‐IL‐1β antibodies significantly decreased the frequency of the granzyme B^+^‐ and perforin^+^ PD1^+^CD8^+^ T cells in tumors of mice bearing the Arf1‐ablated tumor cells (Figure 6i,k and Figure S12e, Supporting Information), indicating that the type I IFNs and IL‐1β cytokines are responsible for enhanced tumor killing ability of the CD8^+^ T cells in mice bearing the Arf1‐ablated tumor cells. These data together suggest that the type I IFNs and IL‐1β cytokines produced by the Arf1‐ablation‐stimulated DCs are responsible for enhancing both the stem cell‐like and effective CD8^+^T cells for anti‐tumor immunity.

## 3 Discussion

**Figure 7:**
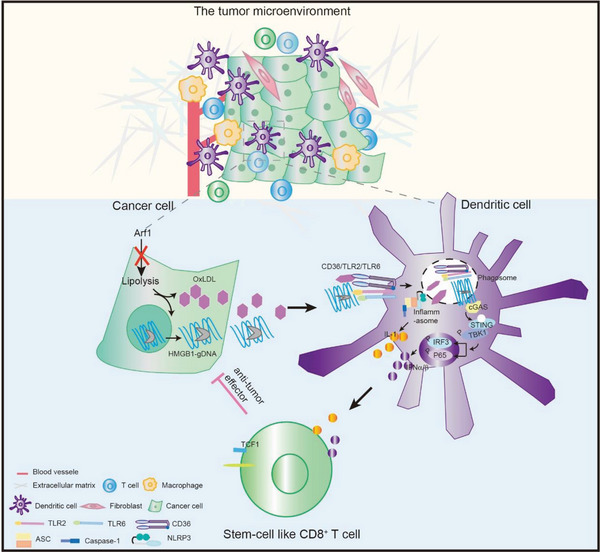
A proposed model of anti‐tumor immunity induced by the Arf1‐ablated tumor cells. Schematic overview of the mechanism: the Arf1‐ablated tumor cells first activate DCs by forming the super signaling complex, which then secret cytokines and chemokines to prime and activate the anti‐tumor CD8^+^T cells in the tumor microenvironment.

It was found that the stem cell‐like CD8^+^ T cells maintain superior responses of ICBs and ACT in animal tumor models and tumor patients. However, the molecular mechanisms by which the immunotherapies regulate the stem cell‐like T cells remain unclear. We recently found that ablation of the small GTPase Arf1 in tumor cells increased T‐cell infiltration and resulted in robust anti‐tumor effects in multiple mouse tumor models that are classically recalcitrant to immunotherapy.^[^ ^27^ ^]^ In this study, we identified a mechanism that mediates the Arf1‐ablation‐induced anti‐tumor immunity. We found that the Arf1‐ablated tumor cells release oxLDL, HMGB1‐gDNA complex, and also induce expression of CD36, TLR2, and TLR6 in DCs. The CD36/TLR2/TLR6 form a coreceptors complex on DC surface, and the exogenous ligands of tumor‐derived oxLDL and HMGB1‐gDNA complex join the coreceptors complex through binding CD36 and TLR2/TLR6, respectively. The cargo‐loaded coreceptor complex then was internalized into the Rab7‐marked phagosomes and further recognized by cytosolic sensors of NLRP3 and cGAS, respectively. HMGB1 could induce TLR‐MyD88‐NF‐κB pathway activation to produce CCL5^[^ ^39^ ^]^. Thus, the super signal complex activates the NF‐κB, cGAS‐STING, and NLRP3 inflammasome triple pathways to produce CCL5, type I IFNs, CXCL10, and IL‐1 family cytokines in DCs. These cytokines and chemokines together regulated DC antigen presentation as well as tumor infiltration, activation, and stemness of T cells, these together promoted a full‐scale robust antitumor immunity (**Figure** **7**).

HMGB1 released by dying tumor cells can bind Toll‐like receptors (TLRs) on DCs and signals through the TLRs‐MyD88‐NF‐κB pathway to induce expression of NLRP3 and pro‐IL‐1β/18 or modifications of NLRP3 for NLRP3 inflammasome priming^[^ ^47^, ^48^ ^]^ as well as efficient processing and cross‐presentation of antigen from dying tumor cells.^[^ ^49^, ^50^ ^]^ Consistent with these findings, we found that the tumor‐derived HMGB1‐gDNA complex preferentially bound to TLR2 receptor, blockage of HMGB1 resulted in reduced protein levels of p65 phosphorylation and NLRP3, as well as significantly decreased production of *Il1b* and *Il18* in DCs (Figure 2g,h and Figure S6b, Supporting Information).

IL‐1β released through the NLRP3 inflammasome pathway can activate antitumor immunity through several mechanisms. First, the released IL‐1β autologously acts on specific DC subsets and second bystander DCs, contributing to their initial maturation, migration, and accumulation in lymphoid organs; second, the DC‐released IL‐1β acts on CD4^+^ T cells to upregulate CD40L expression, which interacts with CD40 on the DC surface to equip the APC for successful CD8^+^ T cell priming; third, IL‐1β can directly polarize CD8^+^ T cell immune responses by acting at the transcriptional level of T cells and also enhance the infiltration of CD8^+^ T cells in the tumor.^[^ ^44^, ^45^ ^]^ We found that activated DCs had enhanced capacity for cross‐priming tumor antigen‐specific CD8^+^ T cells, whereas blockage of DCs activation or depletion of the DC‐derived IL‐1β markedly prevented cross‐priming of CD8^+^T cells and reduced the number of the stemness CD8^+^T cells in the tumor microenvironment (Figures 5 and 6c,d). We also observed that the depletion of IL‐1β cytokine and type I IFNs abrogated tumor regression induced by the Arf1‐ablated tumor cells in mice (Figure 6a,b).

The type I IFNs induced by the cGAS‐SING‐IRF pathway can upregulate chemokine CXCL10 by binding to IFNα and IFNβ receptors (IFNARs) on neoplastic cells, CXCL10 then binds CXCR3 receptor on T cells to increase tumor‐infiltrating T lymphocytes and exerts anti‐tumor effects.^[^ ^52^ ^]^ It was also recently reported that genomic DNA (gDNA) fragments are enriched in the cytosol of CD8^+^ T cells encountered with stimulators and that may activate the cGAS‐STING‐IRF3 pathway to induce type I IFNs, which further inhibit Akt signaling and promote TCF1 expression for maintenance of the stem cell‐like CD8^+^ T cells.^[^ ^53^ ^]^ In the current study, we demonstrated that the tumor gDNA entered into the co‐cultured DCs and directly bound to and activated the cytosolic double‐strand DNA sensor cGAS. The activation of the cGAS‐STING pathway enhanced the production of type I IFNs and chemokine CXCL10. Blockage of the HMGB1‐gDNA significantly prevented activation of the cGAS‐STING signaling pathway and expression of type I IFNs and chemokine CXCL10 in the Arf1‐ablation‐stimulated DCs (Figure 2e,f and Figure S6a, Supporting Information). Importantly, knocking down cGAS or STING in DCs reduced the cross‐priming capacity of DCs for tumor antigen‐specific CD8^+^ T cells. Moreover, we demonstrated that systemic depletion of both type I IFNs and IL‐1β cytokine abrogated tumor regression in mice bearing the Arf1‐ablated tumor cells. These results together clearly demonstrated that the cGAS‐STING pathway‐induced Type I IFNs have critical roles in anti‐tumor immunity induced by the Arf1‐ablated tumor cells.

In summary, in this study, we revealed a new molecular mechanism by which dying tumor cells induce anti‐tumor immune response through releasing several factors that activate the triple pathways in DC for releasing multiple cytokines and chemokines to simultaneously promote DC activation, T cell infiltration, cross‐priming, and stemness. Our findings point out an avenue to enhance immunotherapies of ICBs, ACT, and CAR‐T cells through inhibiting Arf1 or stimulating the super complex signaling pathways. In addition, the Arf1 inhibitors alone demonstrated superior anti‐tumor immunity in animal tumor models and are good candidates to conduct relevant clinical trials in tumor patients.^[^ ^27^ ^]^

## 4 Experimental Section

### Animal Studies

All animals were maintained under specific pathogen‐free conditions at the Laboratory Animal Center of Institute of Developmental Biology and Molecular Medicine, Fudan University. All animal experiments were performed in accordance with the animal study protocols approved by the Animal Care and Use Committee of Fudan University. C57BL/6 and BALB/c mice were originally obtained from Gempharmatech Laboratory Animal Co., Ltd. (Jiangsu, China). C57BL/6 mice were 4–6 weeks of age for preparing bone marrow‐derived dendritic cells. Age‐ and sex‐matched BALB/c mice were 6–8 weeks of age for rechallenging studies.

### Cell Lines

CT26, DLD‐1, and HEK‐293T cell lines were purchased from the American Type Culture Collection (ATCC). DC2.4 dendritic cell was kindly provided by Prof. Penghui Zhou (State Key Laboratory of Oncology in Southern China, Collaborative Innovation Center for Cancer Medicine, Sun Yat‐sen University Cancer Center, Guangzhou, China). CT26 and HEK‐293T cells were cultured in DMEM, supplemented with 10% FBS (HyClone) and 1% penicillin and streptomycin (GIBCO); DLD1 and DC2.4 dendritic cells were cultured in RPMI 1640 medium with 10% FBS and 1% penicillin and streptomycin. All cell lines were maintained at 37 °C with 5% CO2 in a humidified atmosphere. All cell lines were regularly tested for mycoplasma contamination by PCR‐based method and were found to be negative.

### Primary Cell Isolation and Culture

Bone marrow‐derived dendritic cells (BMDCs) were prepared according to the published procedure.^[^ ^54^, ^55^ ^]^ In brief, tibias and femurs were dissected and the attached tissues were removed carefully. The inside of a bone was rinsed to a sterile Petri dish with 5 mL of RPMI 1640 medium without serum but with 2% penicillin and streptomycin (GIBCO). Single‐cell suspension was prepared by first pipetting up and down and then collected in 15 mL centrifuge tubes. Washing twice with RPMI 1640 medium by 10 min centrifugation at 1100 rpm in a refrigerated centrifuge with a swinging bucket rotor. After eliminating red blood cells with 2 mL ACK buffer for 2–5 min at room temperature, the samples were resuspended in 10 mL of RPMI 1640 medium with 10% serum (HyClone) and re‐collected by centrifugation at 1100 rpm for 10 min at 4 °C. Washing twice with the same medium again and centrifugation. After the last washing, the cells were resuspended by 10 mL RPMI 1640 medium supplemented with 10% serum, 20 ng mL^−1^ rm‐GM‐CSF (Cat#CK02, Novoprotein), and 10 ng mL^−1^ rm‐IL‐4 (Cat# 404‐ML, R&D System) and the number of cells were counted. The cell numbers were adjusted to 2 × 10^5^ cells mL^−1^ with the above complete BMDC culture medium. The cells were placed in 10 cm Petri dishes and cultured in a CO2 incubator. Fresh medium with serum and cytokines was added to the cultured cells on day 3. After renewing half of the medium at day 6, the cells were cultured for another 2 days. The loosely adherent cells were collected and BMDCs were sorted by flow cytometry to use on days 8–10.

Naïve CD8^+^T cells were isolated from the spleens of 6‐ to 8‐week‐old OT1 mice. Single‐cell suspensions were obtained by mechanical disruption over a 40 µm cell strainer in a 6 cm cell culture dish contained with 3 mL RPMI 1640 medium. The CD8^+^ T cell selection was carried out with a negative CD8 isolation kit (Cat# 19858A, Stemcell Technologies) following the manufacturer's instructions.

### Cell Culture and Experimental Treatment

The shRNA‐targeted sequences against Arf1, cGAS, Sting,Il1b,Ifna, and Ifnb were synthesized by Sangon Biotech (Shanghai, China). The sequence of shRNAs was as follows:

shScramble:CCGGCTTACGCTGAGTACTTCGACTCGAGTCGAAGTACTCAGCGTAAGTTTTTG;mshArf11#:CCGGGATGCTGTTCTCTTGGTGTTTCTCGAGAAACACCAAGAGAACAGCATCTTTTTG;mshArf12#:CCGGGGAATATCTTTGCAAACCTCTCTCGAGAGAGGTTTGCAAAGATATTCCTTTTTG;hshArf11#:CCGGGAAGACCACGATCCTCTACAACTCGAGTTGTAGAGGATCGTGGTCTTC TTTTTG;hshArf12#:CCGGGCACTCACTACGCCACAGGAACTCGAGTTCCTGTGGCGTAGTGAGTGCTTTTTG;mshCgas1#:CCGGGCCTATTAGTACCAAAGAAGGCTCGAGCCTTCTTTGGTACTAATAGGCTTTTTG;mshCgas2#:CCGGGGAAATCCGCTGAGTCATTTCCTCGAGGAAATGACTCAGCGGATTTCCTTTTTG;

mshSting1#:CCGGGGTACTTGCGGTTGATCTTACCTCGAGGTAAGATCAACCGCAAGTACCTTTTTG;mshSting2#:CCGGGGCAAAGGATCCACCAAATCACTCGAGTGATTTGGTGGATCCTTTGCCTTTTTG;mshIl1b#1:CCGGGCAACCACTTACCTATTTATTCTCGAGAATAAATAGGTAAGTGGTTGCTTTTTG;mshIl1b#2:CCGGCCTCAAAGGAAAGAATCTATACTCGAGTATAGATTCTTTCCTTTGAGGTTTTTG mshIfna#1:CCGGCCTGAACATCTTCACATCAAACTCGAGTTTGATGTGAAGATGTTCAGGTTTTTG;mshIfna#2:CCGGGCTGGCTGTGAGGAAATACTTCTCGAGAAGTATTTCCTCACAGCCAGCTTTTTG;mshIfnb#1:CCGGTGTCAGTTGATGCCTCAGAATCTCGAGATTCTGAGGCATCAACTGACATTTTTG;mshIfnb#2:CCGGGCTCCAAGAAAGGACGAACATCTCGAGATGTTCGTCCTTTCTTGGAGCTTTTTG. Oligo pairs were annealed and subcloned into the polylinker region of the pLKO.1 vector. Lentivirus was produced by HEK293T cells that were co‐transfected with the targeted shRNA in the vector pLKO.1, D8.9 and VSVG plasmids using polyethylenimine (PEI). Viral supernatants were collected at 48–72 h post‐transfection. After centrifugation, the viral supernatants were diluted with fresh complete medium containing 8 µg mL^−1^ polybrene and directly added into the cultured target cells. The established target cells were cultured and selected in DMEM with 10% FBS and 2–10 µg mL^−1^ puromycin for at least 7 days. In some experiments, CRISPR/Cas9 system was used to knockout *Nlrp3*, *Asc*, *caspase‐1*, *Aim2*, *cGAS*, *Sting*, *Cd36*, *Tlr2*, or *Tlr6* genes in DC2.4 cells. The sequence of sgRNAs was as follows: sg*Nlrp3* Forward GACGAGTGTCCGTTGCAAGC, sg*Nlrp3* Reverse GCTTGCAACGGACACTCGTC; sg*Asc* Forward CTATGGGCGCATCCCACGCG, sg*Asc* Reverse CGCGTGGGATGCGCCCATAG; sg*caspase1* Forward AATGAAGACTGCTACCTGGC,sg*caspase1*ReverseGCCAGGTAGCAGTCTTCATT; sg*Aim2* Forward TGCCAGGAGCACACTCGACG, sg*Aim2* Reverse CGTCGAGTGTGCTCCTGGCA; sg*Cd36* Forward TTAATCATGTCGCAATAGCT, sg*Cd36* Reverse AGCTATTGCGACATGATTAA; sg*Tlr2* Forward GGAGGTTCGCACACGCTCGG, sg*Tlr2* Reverse CCGAGCGTGTGCGAACCTCC; sg*Tlr6* Forward GATGCCTCAGGCTCGCCATA, sg*Tlr6* Reverse TATGGCGAGCCTGAGGCATC;sg*Cgas*1#ForwardCCGAGGCGCGGAAAGTCGTA, sg*Cgas*1# Reverse TACGACTTTCCGCGCCTCGG; sg*Cgas*2#Forward AAAGGGGGGCTCGATCGCGG,sg*Cgas*2#ReverseCCGCGATCGAGCCCCCCTTT; sg*Sting*1#ForwardCAGTAGTCCAAGTTCGTGCG,sg*Sting*1#ReverseCGCACGAACTTGGACTACTG;sg*Sting*2#ForwardCACCTAGCCTCGCACGAACT,sg*Sting*2#Reverse AGTTCGTGCGAGGCTAGGTG. Non‐targeting gRNA was used as a negative control. Lipofectamine 2000 (Cat#11668030, Thermofisher) was used for transfecting target cells according to the manufacturer's instructions.

For the non‐contact cell coculture experiments, the trans‐well cell culture plates with 0.4 µM hole diameter (#3412, Corning) were used, and the experimental work model was clearly shown in Figure S1b (Supporting Information), and described as follows: dendritic cells were seeded at the bottom of the trans‐well plates at the concentration of 7 × 10^5^ cells and cultured overnight. After washing twice with warm PBS, 1.5 mL of serum‐free RPMI 1640 (#PM150110, Pricella) medium was added to the cell culture wells. Tumor cells with or without the silence of Arf1 were trypsin digested and washed with warm PBS for twice. After cell number counting, the intended total cells were resuspended in appropriate volume with serum‐free RPMI 1640 medium, then 7 × 10^4^ cells in 500 uL RPMI 1640 medium were added to the upper chamber and cultured overnight. After washing with warm PBS for three times, the upper chamber seeded tumor cells were cocultured with prepared dendritic cells for 24 h, then dendritic cells were collected for experiments. In some experiments, the medium contained with or without cGAS inhibitor RU.521 (Cat#S6841, Selleckchem, 3 µM), STING inhibitor H‐151 (Cat# S6652, Selleckchem, 4 µM), CD36 inhibitor SSO (Cat#SML2148, Sigma Aldrich, 20 µM), or TLR2/6 inhibitors C29 (Cat#HY‐100461, Sigma Aldrich, 50 µM) or MMG‐11 (Cat#HY‐112146, Sigma Aldrich, 50 µM) was used for coculture.

After culturing for 7 days, the BMDCs were collected and seeded in 6‐well plates at a concentration of 2–3 × 10^6^ cells per well. In some experiments, the siRNAs against *cGAS* or *Sting* were added into the medium at a final concentration of 100 nM for 48 h. siRNAs were purchased from Sangon Biotech (Shanghai, China), and the sequence of the siRNAs is as follows: siCgas1# TGGACAAATTGAGATTGAAACGCAA; siCgas2#CAGGTGCTTTCTATCTTGTGAAATT;siSting1#CAGAGGTCACCGCTCCAAATATGTA; siSting2# AGGATCCACCAAATCACACTCTGAA. After washing with warmed PBS for twice, the same number of tumor cells were added into the wells and incubated for another 24 h. Subsequently, CD11c^+^ cells purified by FCS sorting were re‐seeded in 96‐well plates at a concentration of 2 × 10^4^ cells per well, and incubated with isolated CD8^+^T cells from naïve OT1 mice with EasySep Mouse CD8a Positive Selection Kit (Cat# 19858A, Stemcell Technologies) for 3 days at the ratio of 1:10. For stem‐like T cell experiment, tumor cell challenged DC2.4 cells were incubated with isolated CD8+ T cells for 5–10days at the ration of 1:10, with adding fresh medium every two days.

### Tumor Study

CT26 cells (3 × 10^5^) that were ablated with either shScramble or shArf1 were subcutaneously injected into the flank of BALB/C mice. In some experiments, anti‐IFNAR1(Cat#BE0241, Bioxcell) and anti‐IL‐1β (Cat#BE0246, Bioxcell) or appropriate isotype control hamster immunoglobulin and rat IgG2a (Cat# BE0290, Bioxcell) at 8 mg k^−1^g intratumorally were injected on days 0, 2 and 4 after tumor implantations. After tumor cells were injected for 1 week, tumor volumes were determined by manual calipers every day until mice were killed. Tumor volumes were analyzed by length (a) and width (b) and calculated as tumor volume = *ab* ^2^/2. After measurement of tumor weight of individual mouse, the tumors were further analyzed for immune phenotypes.

### Flow Cytometry

Tumors were mechanically disaggregated and single‐cell suspension was obtained using a 70 µm cell strainer. After washing, cells were blocked with anti‐mouse CD16/32 (Cat# AF1960, R&D System) antibody for 20 min and then stained with antibodies targeted to cell surface markers CD3 (172A), CD4 (RM4‐5), CD8 (53‐6.7), TCF1(C63D9), PD‐1 (29F.1A12), H2K^b^ bound to SIINEFKL (25‐D1.16), TIM3 (5D12), CD11c (N418), F4/80 (BM8), Ly6C (HK1.4), CD45 (30‐F11) and CD11b (M1/70) for 45 min at 4 °C. Dead cells were excluded using Live/Dead (1:1000, BioLegend) added concurrently with surface antibodies that were purchased from Biolegend or BD Biosciences. For intracellular cytokine staining, cells were stimulated with phorbol‐12‐myristate 13‐acetate (PMA) (Cat#P1585, Sigma Aldrich, 50 ng mL^−1^) and ionomycin (Cat#S1672, Beyotime, 1 µg mL^−1^) in the presence of Golgi Plug (Cat# 555029, BD Biosciences) and Golgi Stop (Cat#554724, BD Biosciences) for 4 h before cytokine or another antibody staining. Following fixation and permeabilization (Cat# 88–8824, eBioscience), staining was performed with the following antibodies purchased from Biolegend: Ki67 (11F6), T‐bet (4B10), TNF (MP6‐XT22), IFN‐γ (XMG‐1.2), granzyme B (GB11), and Perforin (S16009A). Results were collected on a BD Fortessa and analyzed with FlowJo 10.0 software.

### Western Blot Analysis

For immunoblotting, total proteins were extracted from cells using RIPA lysis buffer supplemented with protease and phosphatase inhibitors (Cat#78446, ThermoFisher, 1:100). Samples were separated on 8%–10% SDS‐PAGE gels and transferred onto PVDF membranes (Cat#IPVH00010, Millipore). Membranes were blocked in 5% BSA for at least 1 hour at room temperature and then incubated in primary antibodies overnight at 4°C. After washing with TBST for 30 minutes, the membranes were incubated with HRP‐conjugated secondary antibody for one hour at room temperature. After washing again, signals were enhanced by chemiluminescence (Cat#34577, Thermo Fisher). The primary antibodies are GAPDH (Cat# HRP‐60004, Proteintech; 1:50 000), NLRP3 (Cat# 15101, Cell Signaling Technology, 1: 3000), IL‐1β (Cat# 12242, Cell Signaling Technology, 1: 1000), caspase‐1(Cat# sc‐398715, Santa Cruz Biotechnology, 1: 500), cGAS (Cat# 31659, Cell Signaling Technology, 1: 2000), STING (Cat# 13647, Cell Signaling Technology, 1: 2000), TBK1 (Cat# 3504, Cell Signaling Technology, 1: 2000), p‐TBK1 (Cat# 5483, Cell Signaling Technology, 1: 1000), P65(Cat# 8242, Cell Signaling Technology, 1: 2000), p‐P65(Cat# 3033, Cell Signaling Technology, 1: 1000), IRF3 (Cat# 4302, Cell Signaling Technology, 1: 2000), p‐IRF3 (Cat# 29047, Cell Signaling Technology, 1: 1000), p‐STAT2 (Cat# AP0284, ABclonal, 1:500), TLR6 (Cat# 12717, Cell Signaling Technology, 1: 1000), TLR2 (Cat# AB209217, Abcam, 1:2000), TLR4 (Cat# 14358, Cell Signaling Technology, 1: 1000), CD36 (Cat# ab252923, Abcam, 1:1000), HMGB1 (Cat# MA5‐31967, ThermoFisher, 1:1000), Rab7 (Cat# 9367, Cell Signaling Technology, 1: 1000).

### Immunofluorescent Staining

For cell immunofluorescent staining, tumor cells or DCs were seeded on 20‐ or 25‐mm diameter glass slides placed in a 12‐ or 6‐well plate. After serum starvation overnight or transfection, cells were treated with exogenous Dil‐oxLDL(Cat# L34358, ThermoFisher) alone or co‐cultured in the presence or absence of specific inhibitors for 24 hours. 10 mM EdU was added into the culture medium of the tumor cells and then cultured overnight. Cells were fixed with 4% PFA for 30 minutes, then slides were washed and permeabilized, followed by blocking with 10% FBS for one hour and then incubated with the following antibodies overnight at 4°C: anti‐cGAS (Cat# 31659, Cell Signaling Technology, 1:200), anti‐HMGB1 (Cat# MA5‐31967, ThermoFisher, 1:100), anti‐ASC (Cat# 67824, Cell Signaling Technology, 1:200), anti‐CD36 (Cat# sc‐7309, Santa Cruz Biotechnology, 1:200), anti‐TLR2 (Cat# 14‐9021‐82, ThermoFisher, 1:200), or anti‐TLR6 (Cat# 12717, Cell Signaling Technology, 1:200). Slides were washed with PBS for 30 minutes and incubated with the secondary antibody for one hour at room temperature. EdU staining was performed according to the manufacturer's instructions (Cat# C10339, Thermofisher). Hoechst (Cat#H21492, Thermofisher) was used to visualize cell nuclei. Images were taken with a Zeiss LSM 880 confocal microscope.

### Co‐Immunoprecipitation

After coculture with the Scramble‐ablated or the Arf1‐ablated tumor cells for 24 h, DC2.4 dendritic cells were washed with cooled PBS for 2 times and placed on ice. Then the cells were lysed by incubation for 15 min at 4 °C with NP‐40 lysis buffer (Cat# P0013F, Beyotime) containing proteinase inhibitor cocktail (Cat#78446, ThermoFisher). After centrifugation at 12 600 rpm for 15 min at 4°C, the supernatants were collected to perform immunoprecipitation and incubated with rabbit anti‐mouse CD36 antibody (Cat# sc‐7309, Santa Cruz Biotechnology, 1:100), rat anti‐mouse TLR2 antibody (Cat# 14‐9021‐82, ThermoFisher, 1:100), rabbit anti‐mouse TLR6 antibody (Cat# 12717, Cell Signaling Technology, 1:100) or rabbit anti‐mouse Rab7 antibody (Cat# 9367, Cell Signaling Technology, 1:100) overnight at 4 °C. 20 µL of agarose beads (Cat# P2197, Beyotime) was added and incubated for another 2 h at 4 °C. Antigen‐antibody‐beads complex was collected after centrifugation at 2000 rpm for 3 min and washed with NP‐40 buffer for three times. The complex was resuspended with 80 µL sample loading buffer and boiled for 10 min, followed by immunoblotting analysis.

### Chromatin Immunoprecipitation

The Arf1‐ablation‐stimulated DC2.4 dendritic cells or supernatants of the Arf1‐ablated tumor cells were fixed with 1% formaldehyde. The cells were then lysed and centrifuged for 10 min at 18 000 g in a 4 °C microcentrifuge. The tumor supernatants or the collected cellular lysis was prepared to perform immunoprecipitation and incubated with rabbit anti‐mouse HMGB1 antibody (Cat# MA5‐31967, ThermoFisher, 1:50) or rabbit anti‐mouse cGAS antibody (Cat# 31659, Cell Signaling Technology, 1:50) overnight at 4 °C. 25 µL of Pierce Protein A/G Magnetic Beads (Cat# 88804, ThermoFisher) was added and incubated for another 2 h at 4 °C. Antigen‐antibody‐beads complex was washed with Lysis/Wash buffer for three times and collected after elution with 100 µL Elution Buffer, and 10 µL of Neutralization Buffer was added for each of the eluate. After purification with a chromatin DNA purification kit (Cat# D4002, ZYMO RESEARCH), the DNA was analyzed by qPCR with human or mouse gDNA or mtDNA primer pairs. The fold enrichment of gDNA or mtDNA in HMGB1 or cGAS immunoprecipitates was calculated. The following primer sequences were used:m*Polg1*ForwardGATGAATGGGCCTACCTTGA,m*Polg1*ReverseTGGGGTCCTGTTTCTACAGC;m*Nd1*ForwardCAAACACTTATTACAACCCAAGAACA,m*Nd1*ReverseTCATATTATGGCTATGGGTCAGG.

### Real‐Time Polymerase Chain Reaction

RNAs were extracted from cells using Trizol (Beyotime) and complemental DNA was generated using ReverTra Ace qPCR RT kit (FSQ‐101, TOYOBO) according to the manufacturer's instructions. Real‐time PCR was performed using the 2 × Trans‐Start Top Green qPCR SuperMix (Transgene). The PCR results were normalized to *Gapdh* expression. Primer sequences are as follows: m*Arf1* Forward AGGTCTTTGGCCAGTATC, m*Arf1* Reverse ACATTGAAACCAATGGTGG; h*Arf1* ForwardAACACCTTCGCTGTCTGGGATG,h*Arf1*ReverseGGCAAGTGAGCCTTGATGTGTG; m*Gapdh* Forward TGTGTCCGTCGTGGATCTGA, m*Gapdh* Reverse TTGCTGTTGAAGTCGCAGGAG; *Il6* Forward CAAGAAAGACAAAGCCAG AGTC,*Il6* ReverseGAAATTGGGGTAGGAAGGAC; *Ifnb*ForwardGCCTTTGCCATCCAAGAGATGC, *Ifnb* Reverse ACACTGTCTGCTGGTGGAGTTC; *Il1b* Forward CTGAAGCAGCTATGGCAACTG, *Il1b* Reverse TTTCAGCTCATATGGGTCCGA;*Il18*ForwardGACTCTTGCGTCAACTTCAAGG;*Il18*ReverseCAGGCTGTCTTTTGTCAACGA; *Tnf* Forward CATCTTCTCAAAATTCGAGTGACAA, *Tnf* Reverse TGGGAGTAGACAAGGTACAACCC;*Cxcl10*ForwardTGAACCCAAGTGCTGCCGTC, *Cxcl10* Reverse CATCGTGGCAATGATCTCAA; *Tlr4* Forward ATGGCATGGCTTACACCACC, *Tlr4* Reverse GAGGCCAATTTTGTCTCCACA; *Tlr6*ForwardTGAGCCAAGACAGAAAACCCA, *Tlr6* ReverseGGGACATGAGTAAGGTTCCTGTT; *Tlr2* Forward GCAAACGCTGTTCTGCTCAG, *Tlr2* Reverse AGGCGTCTCCCTCTATTGTATT; *Tlr9* Forward ATGGTTCTCCGTCGAAGGACT, *Tlr9*ReverseGAGGCTTCAGCTCACAGGG;*Cd36*ForwardCTGGGACCATTGGTGATGAAA, *Cd36* Reverse CACCACTCCAATCCCAAGTAAG; *Ccl5* Forward CACCATCATCCTCACTGCAG, *Ccl5* Reverse CTTCGAGTGACAAACACGAC. *Ifna*ForwardGCTTTCCTGATGGTTTTGGTG,*Ifna*ReverseAGGCTTTCTTGTTCCTGAGG;*Ifit1*ForwardCTGAGATGTCACTTCACATGGAA,*Ifit1*ReverseGTGCATCCCCAATGGGTTCT;*RSAD2*ForwardTGCTGGCTGAGAATAGCATTAGG, *RSAD2* Reverse GCTGAGTGCTGTTCCCATCT;

### Enzyme‐Linked Immunosorbent Assay for Cytokines

Cytokines in the supernatants were analyzed by mouse IL‐1β ELISA kit (Cat# E‐EL‐M0037c, Elabscience), and IFNb ELISA kit (Cat# E‐EL‐M0033c, Elabscience) according to the manufacturer's instructions, respectively.

### Cytosolic DNA Extraction and Analysis

After coculture with or without the Arf1‐ablated tumor cells for 24 h, DC2.4 dendritic cells were collected from each well of the 6‐well plates and divided into equal aliquots.^[^ ^42^ ^]^ Then one aliquot was lysed in 100 µL 50 µM NaOH and boiled for 15 min. These whole cell lysates were neutralized with 10 µL of 1 m Tris‐HCl pH 8 (10 ×), which served as normalization controls for total DNA. The other equal aliquots were resuspended in 100 µL cytosolic extract buffer containing 150 mM NaCl, 50 mM HEPES (Cat# 15630080, Gibco), and 25 µg mL^−1^ digitonin (Cat# 300 410, Sigma) and incubated for 10 min on ice for plasma membrane permeabilization. After centrifugation, the cytosolic supernatants were collected and again centrifuged at 13 800 rpm for 10 min to pellet the remaining cellular debris. DNA from whole cell lysates or cytosolic extract was subjected to protease K for 2 h at 58 °C and purified using DNA Clean and Concentrator Kit (Cat# D4003, ZYMO RESEARCH). Quantitative PCR was performed on both whole‐cell extracts and cytosolic fractions using gDNA primers (mouse or human) and mtDNA primers (mouse or human). The mouse primers were listed above, and the following human primers were used: h*Polg1*ForwardGCTGCACGAGCAAATCTTCG,h*Polg1*ReverseGTCCAGGTTGTCCCCGTAGA;h*Nd1*ForwardGGCAGGAGTAATCAGAGGTG,h*Nd1*ReverseAACATACCCATGGCCAACCT.

### Quantification and Statistical Analysis

GraphPad Prism 5.0 software was used for all experimental result analysis. All data were represented as the mean ± standard error of the mean (SEM) or SD. All *n*‐values per group were reported in the figure legends. Statistical significance was represented in figures as follows: *p < 0.05, **p < 0.01, ***p < 0.001, ****p < 0.0001 and n.s. indicates not significant. *p*‐Values were determined by unpaired two‐tailed Student's t‐test or by two‐way ANOVA with Tukey's multiple comparisons test. A *p*‐value that was <0.05 was considered statistically significant. For each quantification by immunofluorescence, at least six macroscopic fields per biological replicate and at least three biological replicates per group were analyzed. In the study, no statistical methods were used to predetermine sample size, and the investigators were not blinded to allocation during experiments and result analysis.

## Conflict of Interest

The author declares no conflicts of interest.

## Author Contributions

H.M., Y.W., and S.X.H. conceived and designed the experiments. H.M., W.F., Q.L., and Y.W. performed the experiments. H.M., Y.W., and S.X.H. analyzed the data. H.M. and S.X.H. wrote the manuscript.

## Supporting information

Supporting Information

## Data Availability

The data that support the findings of this study are available from the corresponding author upon reasonable request.
