# Supplementary material for: Arf1 Ablation in Colorectal Cancer Cells Activates a Super Signal Complex in DC to Enhance Anti‐Tumor Immunity
Source: Adv Sci (Weinh). 2023 Oct 2;10(32):2305089. doi: 10.1002/advs.202305089 (PMC10646219; doi:10.1002/advs.202305089)
Supplement: Supplementary file 1 — Supporting Information [file ADVS-10-2305089-s001.pdf]

ADVANCED SCIENCE
Open Access

Supporting Information

for *Adv. Sci.*, DOI 10.1002/advs.202305089

Arf1 Ablation in Colorectal Cancer Cells Activates a Super Signal Complex in DC to Enhance Anti-Tumor Immunity

Handong Ma, Wanqi Fang, Qiaoming Li, Yuetong Wang and Steven X. Hou**

Supplementary Materials for

Arf1 ablation in colorectal cancer cells activates a super signal complex in DC to enhance anti-tumor immunity

Handong Ma, Wanqi Fang, Qiaoming Li, Yuetong Wang*, Steven X. Hou*

Department of Cell and Developmental Biology at School of Life Sciences, State Key Laboratory of Genetic Engineering, Institute of Metabolism and Integrative Biology, Human Phenome Institute, Department of Liver Surgery and Transplantation of Liver Cancer Institute at Zhongshan Hospital, Fudan University, Shanghai 200438, China

*Correspondence: ytwang@fudan.edu.cn (Y.T.W); stevenhou@fudan.edu.cn (S.X.H.)

Supplementary Figure 1

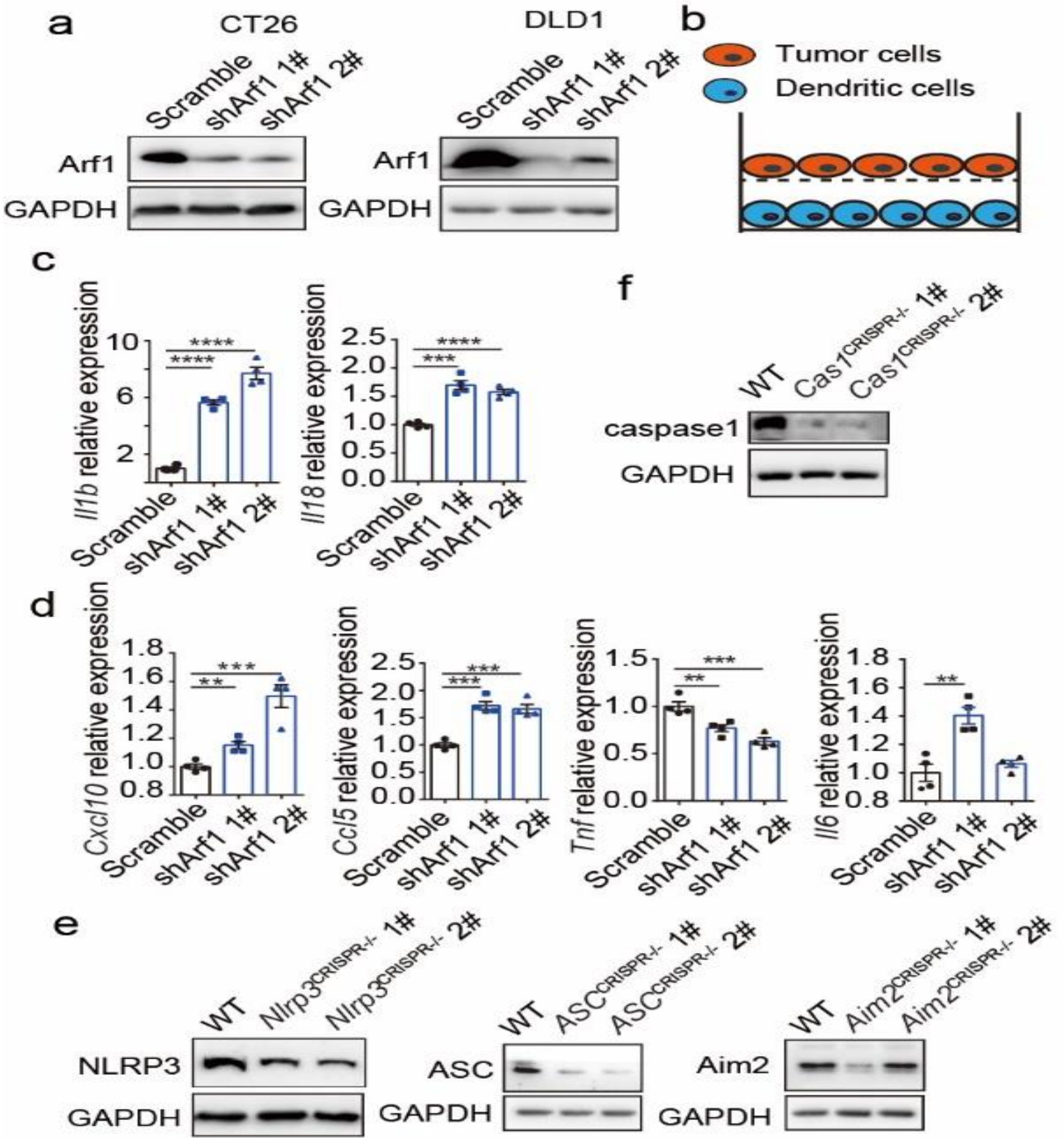

Supplementary Figure 1. Arf1-ablation in tumor cells promotes expression of inflammatory cytokines

a) Immunoblotting analysis of Arf1 and GAPDH in CT26 or DLD1 cancer cells that were transfected with the indicated constructs. **b**) Experimental design: DC2.4 dendritic cells were cocultured with either the Scramble- or Arf1-ablated tumor cells. **c, d**) The expression of indicated genes in DC2.4 cells after coculture with either the Scramble- or Arf1-ablated CT26 cells was performed by RT-qPCR analysis (n=3). **e, f**) DC2.4 dendritic cells were transfected with or without the indicated constructs, and immunoblotting analysis were performed to evaluate the knockout efficiency by CRISPR-Cas9 technology. In all of the panels, data are presented as means ± SEMs; $^*p < 0.05$, $^{**}p < 0.01$, $^{***}p < 0.001$, $^{****}p < 0.0001$. Student's *t* test.

Supplementary Figure 2

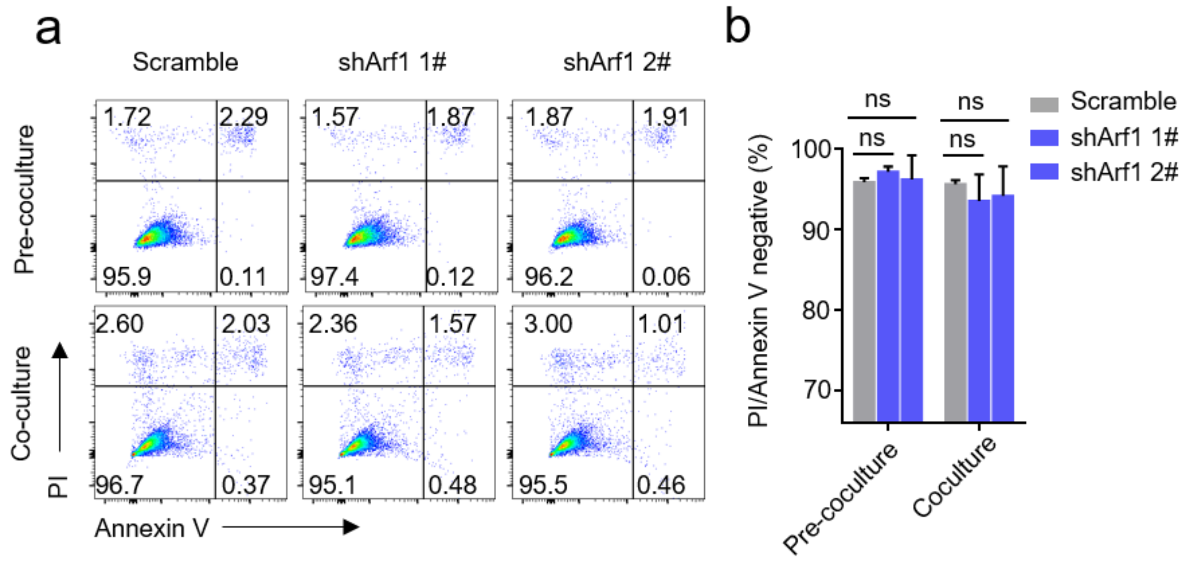

Supplementary Figure 2. Genetic deletion of Arf1 does not alter the proportion of dying tumor cells

a, b) Flow cytometry analysis of dying cells of Arf1-ablation or scramble control tumor cells before and after coculture with dendritic cells for 24 hr (a), and quantification of dying cells (b) as described in (a). n = 3 biological replicates.

Data are from one experiment representative of three independent experiments with similar results. In panel (b) data are presented as means ± SDs; *P* values (not significant) were determined by one-way ANOVA with Tukey's test.

Supplementary Figure 3

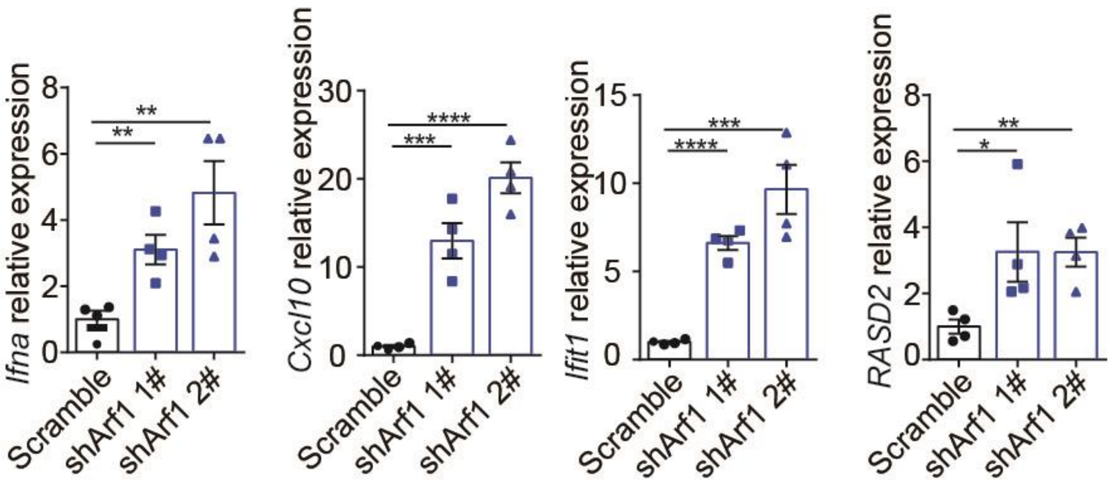

Supplementary Figure 3. The expression of type I IFNs and their regulated genes was enhanced in DCs from tumors of mice transplanted with the Arf1-ablated CT26 cells

Analysis of the expression of *Ifna*, *Cxcl10*, *Ifit1* and *RASD2* in dendritic cells that were isolated from tumors of mice transplanted with either the Scramble- or Arf1-ablated CT26 cells (n=4).

In all of the panels, data are presented as means ± SEMs; *$p < 0.05$, **$p < 0.01$, ***$p < 0.001$, ****$p < 0.0001$. Student's *t* test.

Supplementary Figure 4

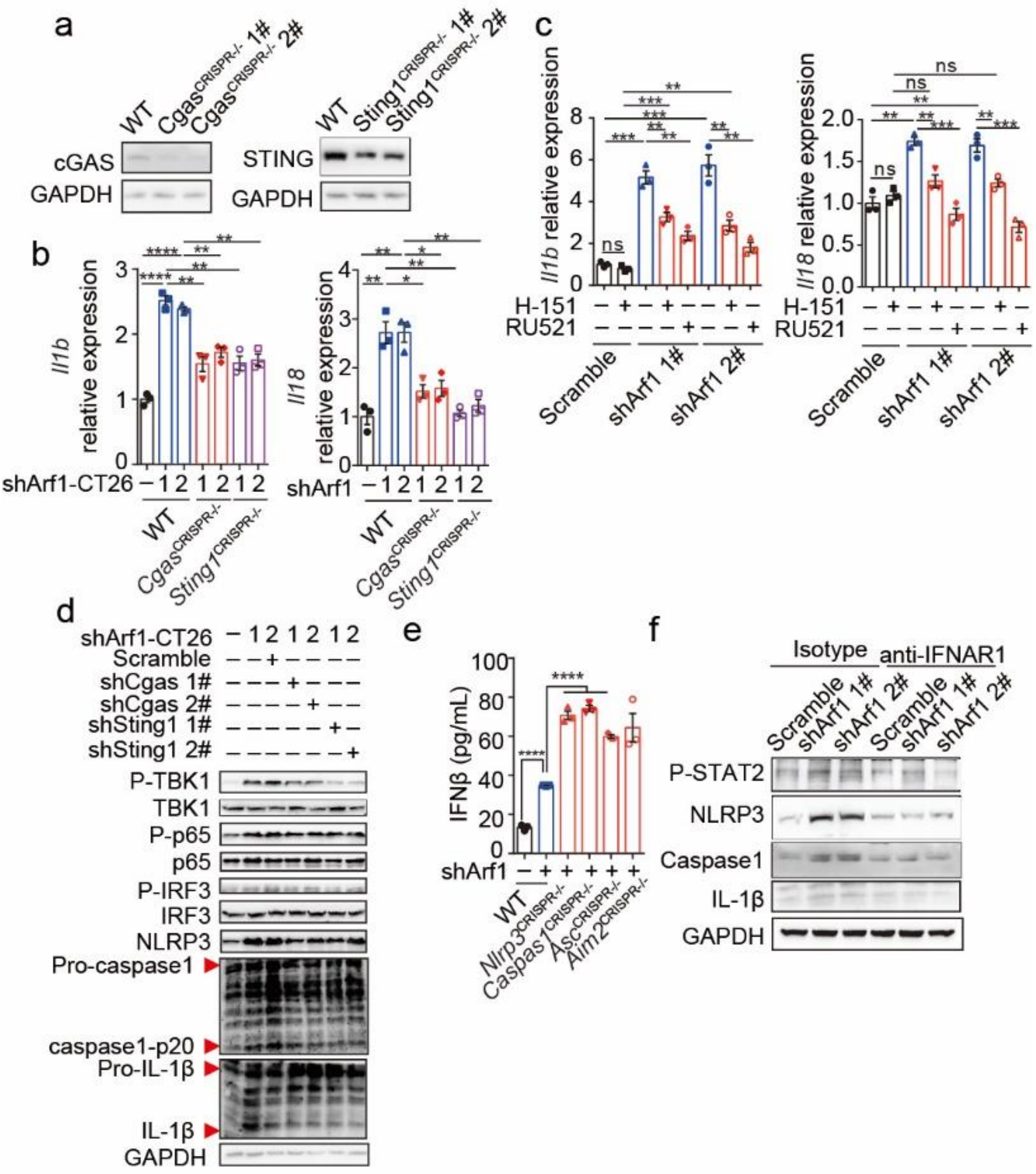

Supplementary Figure 4. The NLRP3 inflammasome activation is downstream of the cGAS-STING and NF-kB signaling pathways

a) Immunoblotting analysis of cGAS and STING in DC2.4 cells that were transfected with or without indicated constructs. GAPDH was used as a loading control. **b**) The

expression level of *Il1b* and *Il18* in DC2.4 cells that were transfected with either the vector or indicated constructs and then cocultured with either the Scramble- or Arf1-ablated CT26 cells (n = 3). 1 indicates shArf1 #1-transfected CT26 cells, 2 indicates shArf1 #2-trasfected CT26 cells. **c**) The expression level of *Il1b* and *Il18* in DC2.4 cells that were cocultured with either the Scramble- or Arf1-ablated CT26 cells in the presence or absence of cGAS inhibitor RU521 or STING inhibitor H151 for 24 hr (n = 3). **d**) Immunoblotting analysis of the indicated proteins in DC2.4 cells that were transfected with either the vector or indicated constructs and then cocultured with either the Scramble- or Arf1-ablated CT26 cells. **e**) Quantification of the secreted IFNβ from DC2.4 cells that were transfected with either the vector or indicated constructs and then cocultured with either the Scramble- or Arf1-ablated CT26 cells (n=3). **f**) Immunoblotting analysis of the indicated proteins in DC2.4 cells that were cocultured with either the Scramble- or Arf1-ablated CT26 cells in the presence or absence of anti-IFNAR1 antibody for 24 hr.

In all of the panels, data are presented as means ± SEMs; *p < 0.05, **p < 0.01, ***p <0.001, ****p < 0.0001. n.s., not significant. Student's *t* test.

Supplementary Figure 5

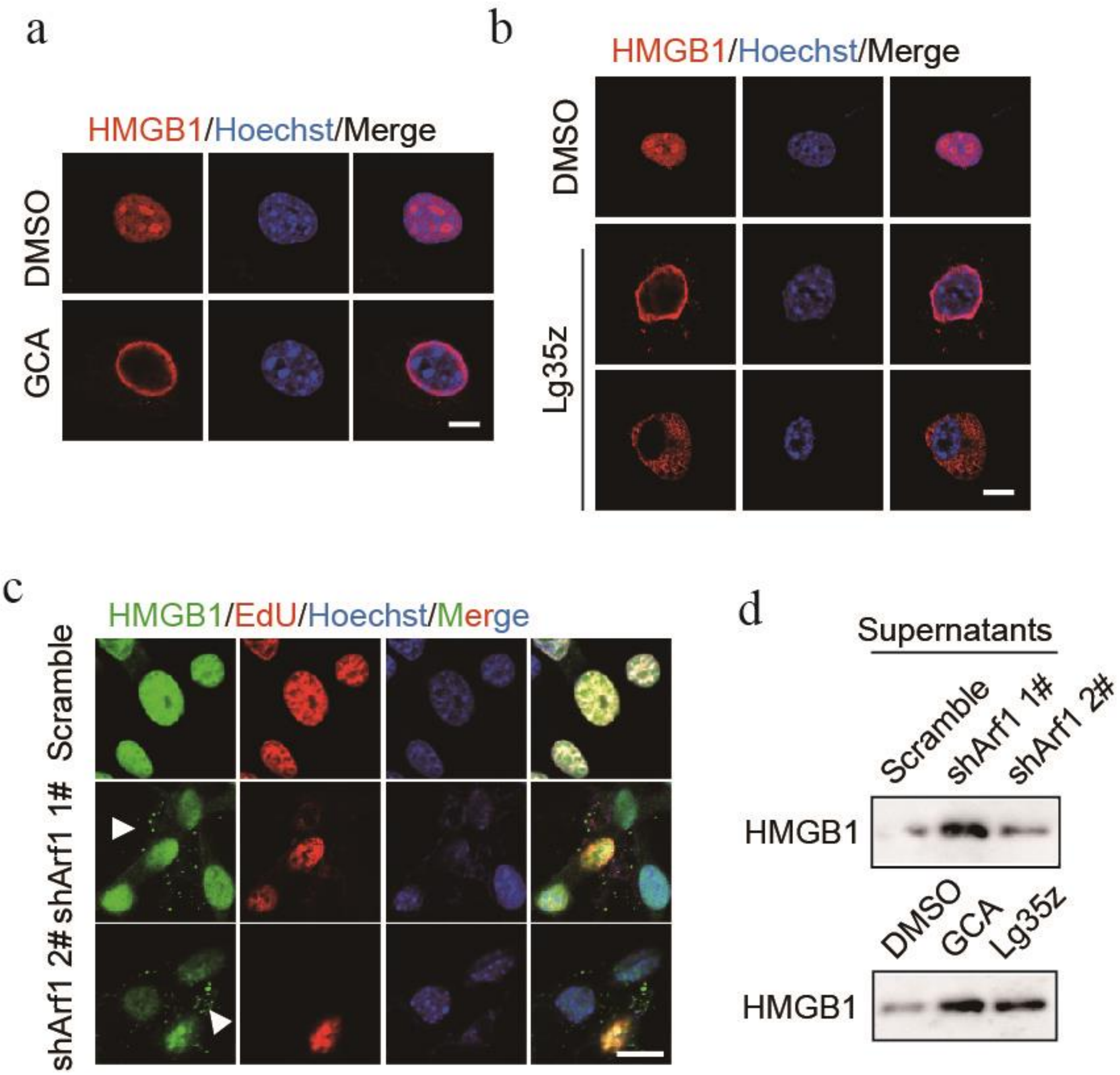

Supplementary Figure 5. Deletion of Arf1 leads to HMGB1/DNA translocation and release

a,b) Immunofluorescence staining of HMGB1 in CT26 tumor cells that were treated with DMSO or Arf1 inhibitors GCA (**a**) or Lg35z (**b**) for 24 hr. Scale bars, 25 μm. **c)** Immunofluorescence staining of HMGB1 and EdU in CT26 cells that were transfected with either the Scramble- or shArf1 and incubated with 10 μM EdU for 24 hr. The arrow indicates the distribution of HMGB1 protein around Arf1-ablated tumor cell(s). Scale bars, 50 μm. **d**) Immunoblotting analysis of HMGB1 protein levels in the supernatants collected from either the Scramble- or Arf1-ablated CT26 cells.

Supplementary Figure 6

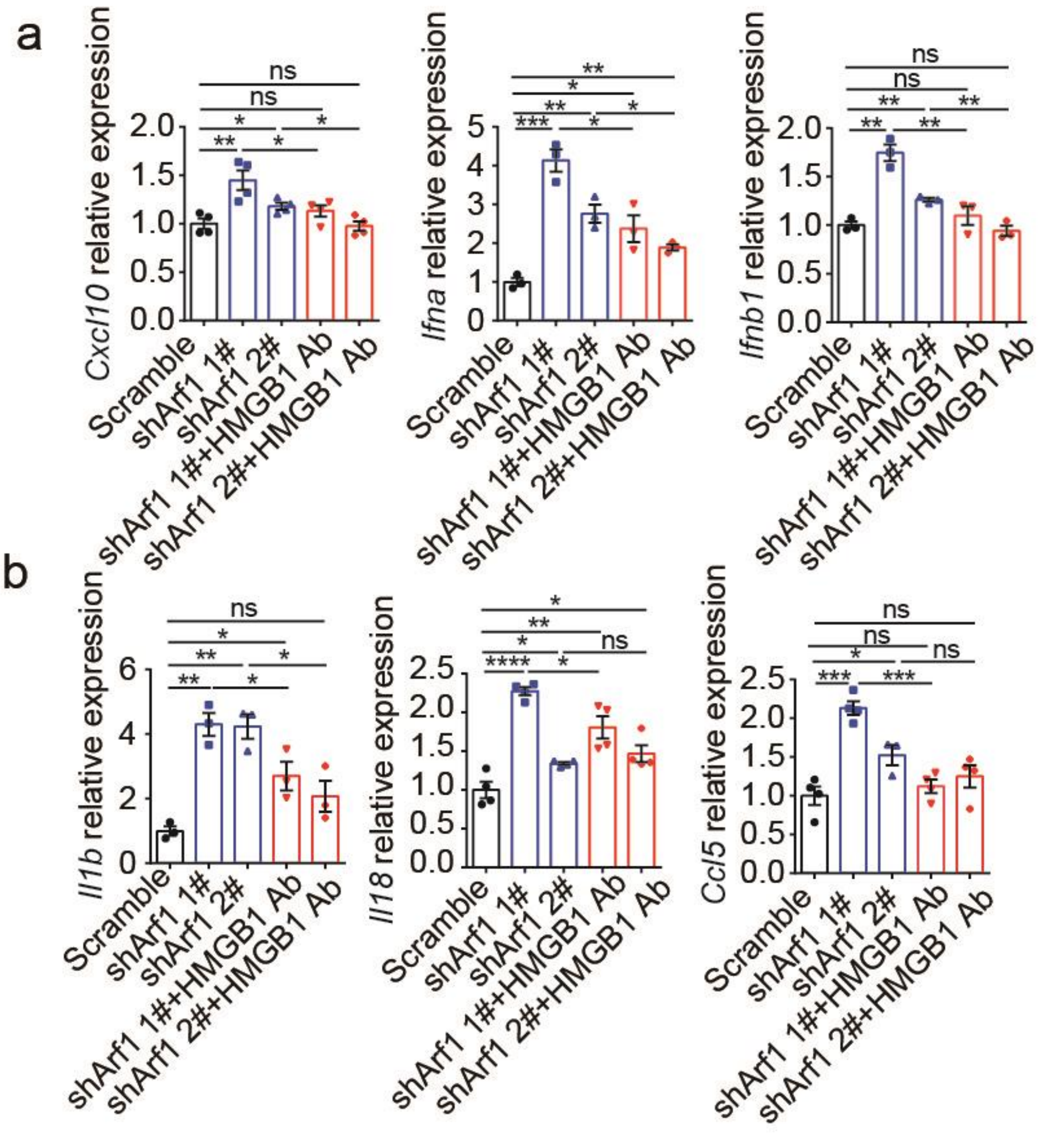

Supplementary Figure 6. Blockage of HMGB1 prevents both the cGAS-STING and NF-kB pathways activation

a) The expression of indicated genes in DC2.4 cells that were cocultured with either the Scramble- or Arf1-ablated CT26 cells in the presence or absence of 5 μg/ml anti-HMGB1 antibody (n = 3). **b**) Analysis of *Il1b*, *Il18* and *Ccl5* expression in DC2.4 cells that were treated as described in (**a**). (n = 3).

In all of the panels, data are presented as means ± SEMs; *p < 0.05, **p < 0.01, ***p <0.001, ****p < 0.0001. n.s., not significant. Student's *t* test.

Supplementary Figure 7

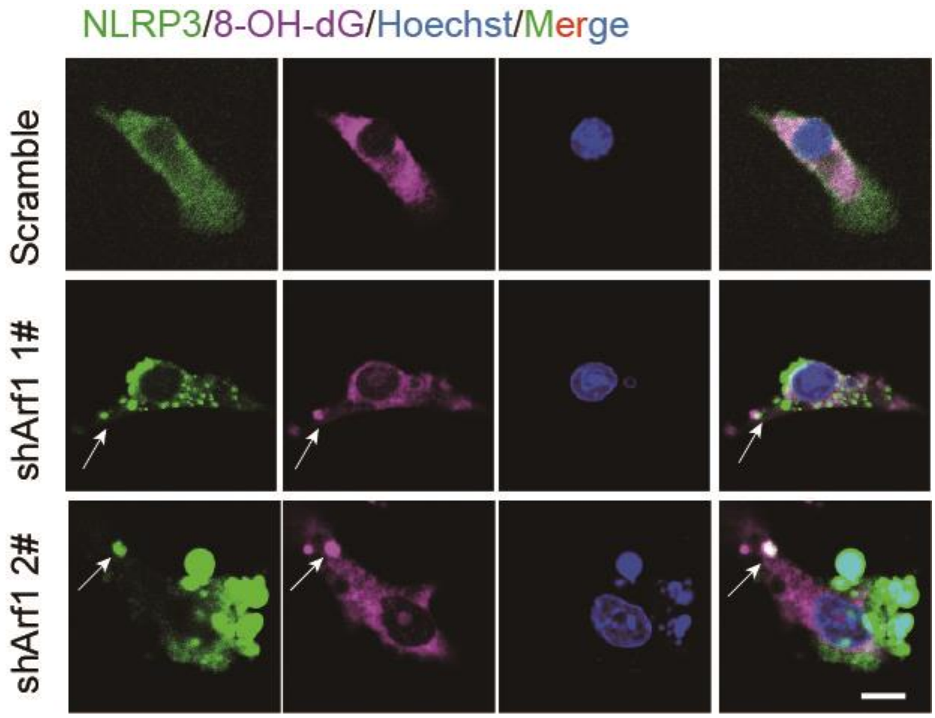

Supplementary Figure 7. NLRP3 colocalized with oxidative DNA from the Arf1-ablated tumor cells

Immunofluorescence staining of NLRP3 and 8-OH-dG in DC2.4 cells after coculture with either the Scramble- or Arf1-ablated CT26 cells for 24 hr. The arrows indicate NLRP3 or 8-OH-dG positive specks and merged specks of these two proteins, respectively. Scale bars, 25 µm

Supplementary Figure 8

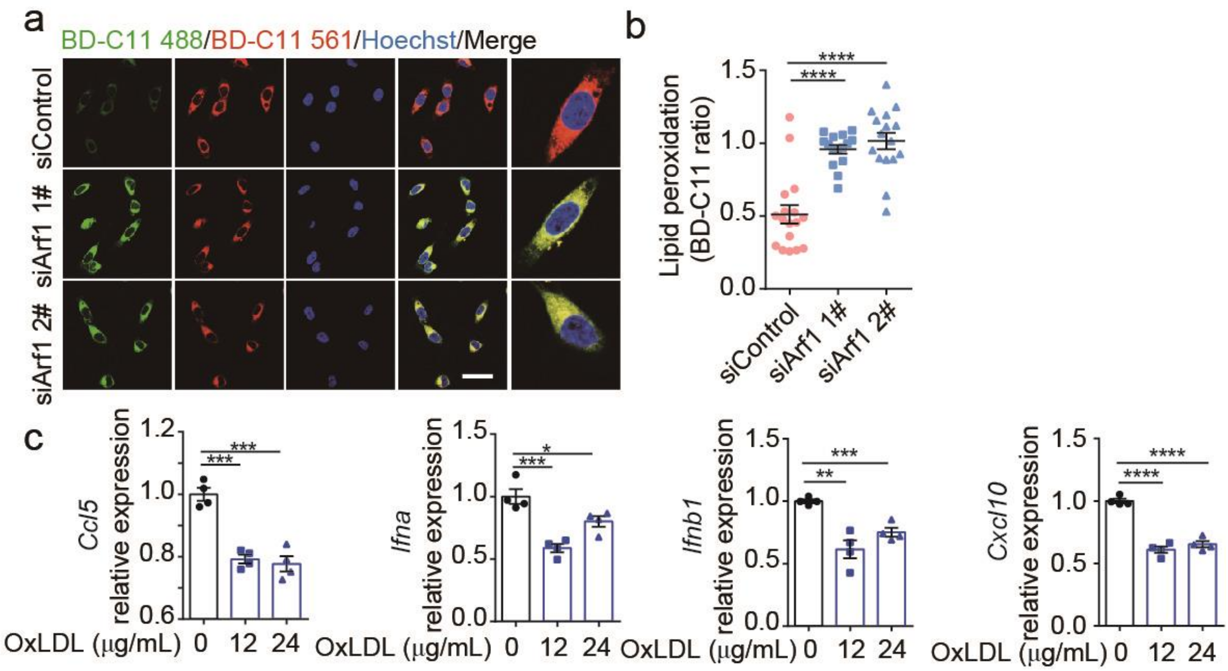

Supplementary Figure 8. The Oxidized lipids do not induce activation of the cGAS-STING and NF-kB pathways

a, b) CT26 cells were transfected with the indicated siRNAs and incubated with 30 μM BODIPY-C11. Fluorescence intensity of 488 nm and 561 nm were detected by confocal (**a**) and the ratio of green to red was quantified (**b**). Scale bars, 50 μm. **c**, Analysis of the expression of indicated genes in DC2.4 cells that were treated with or without the indicated concentrations of OxLDL (n = 4).

Each point represents the ratio of green to red (**b**). In all of the panels, data are presented as means ± SEMs; *p < 0.05, **p < 0.01, ***p <0.001, ****p < 0.0001. Student's *t* test.

Supplementary Figure 9

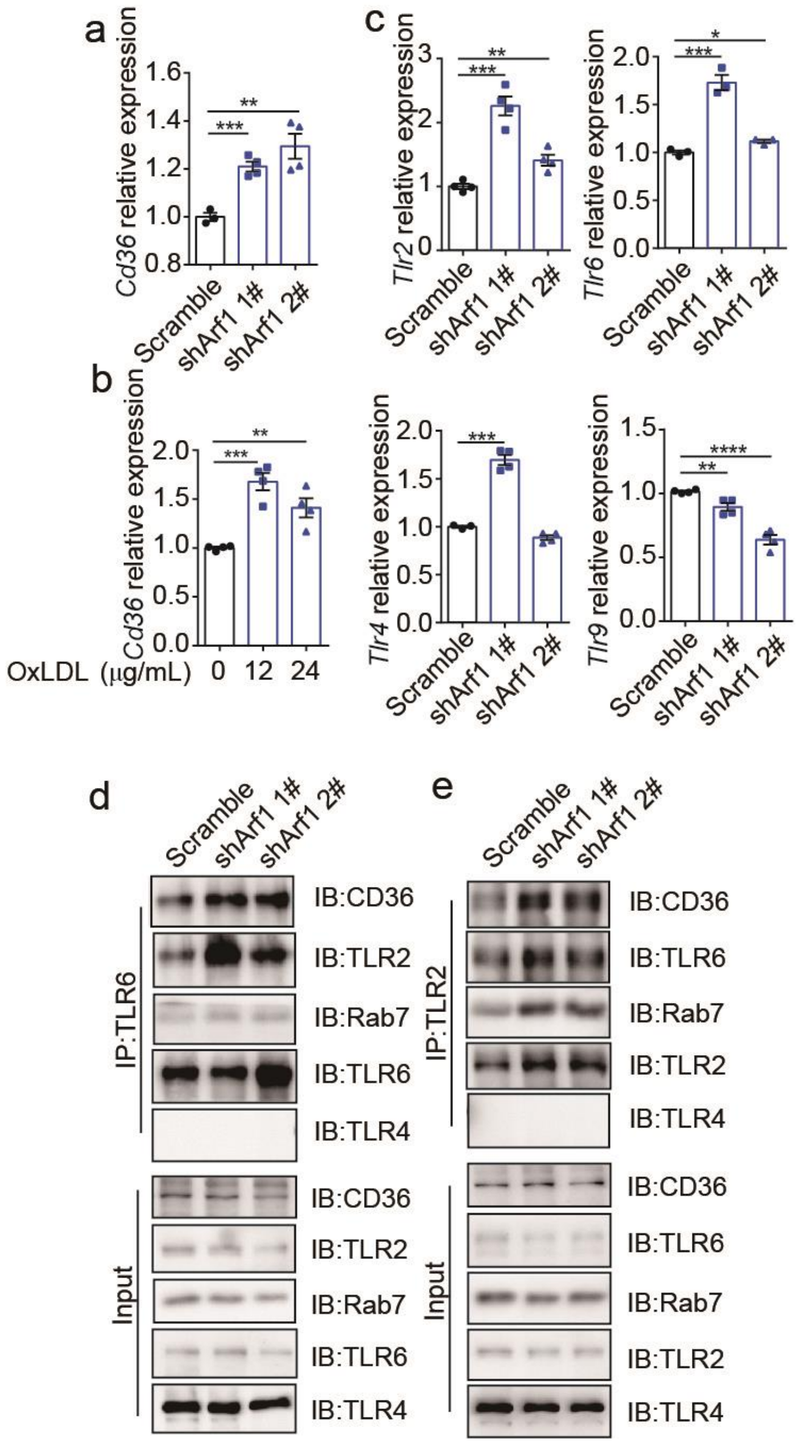

Supplementary Figure 9. The co-receptors (CD36, TLR2, TLR6) were induced by the Arf1-ablated tumor cells and co-localized on the DC surface

a) Analysis of the expression of Cd36 in DC2.4 cells after coculture with either the Scramble- or Arf1-ablated CT26 cells (n = 3-4). **b**) DC2.4 cells were treated with or

without indicated concentrations of OxLDL for 24 hr and the expression of Cd36 was analyzed by RT-qPCR (n = 4). **c**) Analysis of the expression of the indicated genes in DC2.4 cells after coculture with either the Scramble- or Arf1-ablated CT26 cells (n = 4). **d**) Co-immunoprecipitation to detect binding of TLR6 to CD36, TLR2, TLR4 and Rab7 in DC2.4 cells after coculture with either the Scramble- or Arf1-ablated CT26 cells. **e**) Co-immunoprecipitation to detect binding of TLR2 to CD36, TLR6, TLR4 and Rab7 in DC2.4 cells after coculture with either the Scramble- or Arf1-ablated CT26 cells.

In all of the panels, data are presented as means ± SEMs; *p < 0.05, **p < 0.01, ***p <0.001, ****p < 0.0001. Student's *t* test.

Supplementary Figure 10

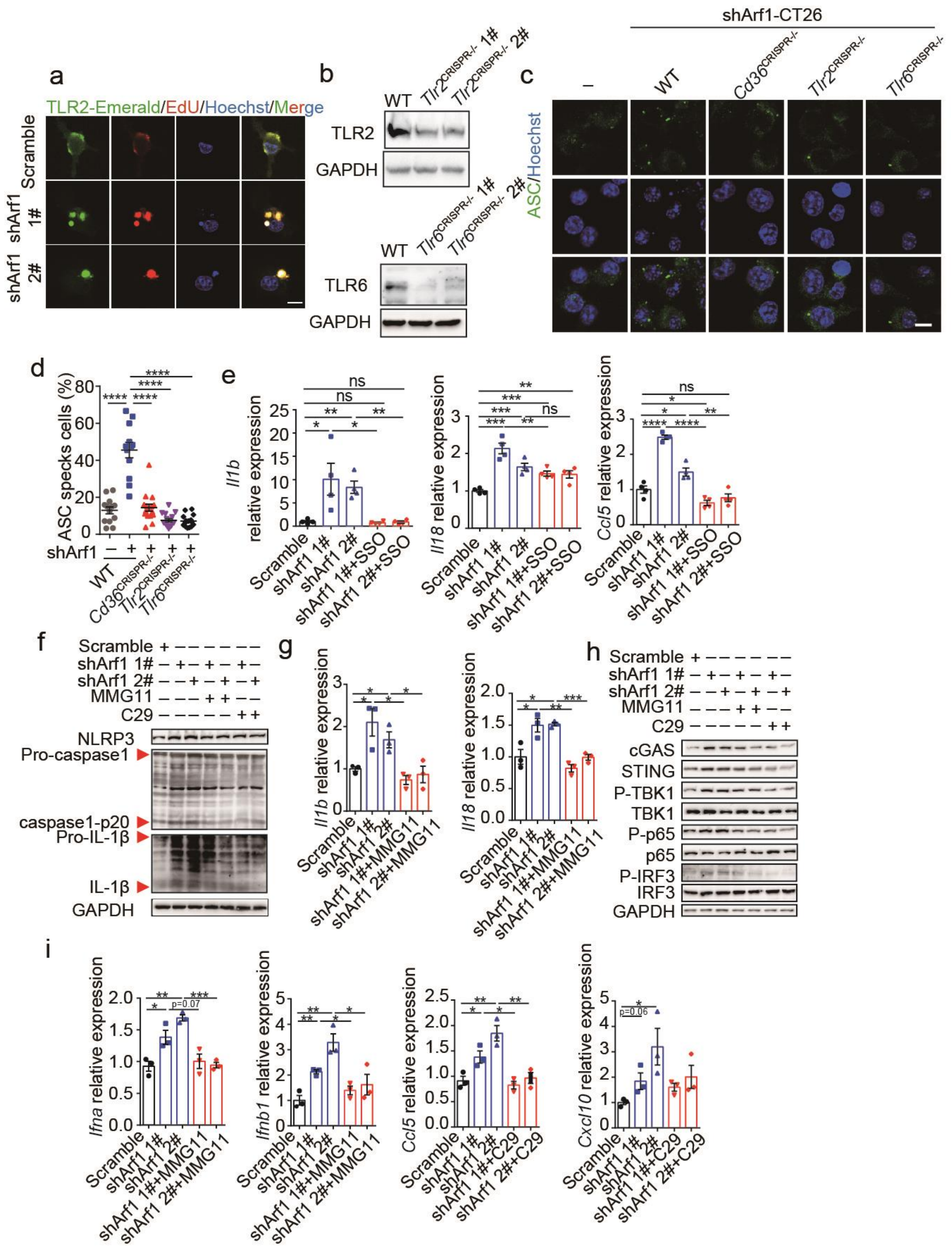

Supplementary Figure 10. Knockdown of CD36, TLR2 or TLR6 prevents activation of the triple pathways induced by the Arf1-abalted tumor cells in DC

a) Immunofluorescence staining of EdU and TLR2 in DC2.4 cells that were transfected with the TLR2-Emerald constructs and then cocultured 10 μM EdU incubated Scramble- or Arf1-ablated CT26 cells. Scale bars, 25 μm. **b**) Analysis of the indicated proteins in DC2.4 cells that transfected with either vector or the indicated constructs for 48 hr. **c, d**) Immunofluorescence staining of ASC (**c**) and quantification of ASC specks (**d**) in DC2.4 cells that were transfected with either the vector or indicated constructs and then cocultured with either the Scramble- or Arf1-ablated CT26 cells (n = 4). Scale bars, 25 μm. **e**) Analysis of the expression of *Il1b*, *Il18* and *Ccl5* in DC2.4 cells that were cocultured with either the Scramble- or Arf1-ablated CT26 cells in the presence or absence of 20 μM SSO for 24 hr (n = 3). **f, h**) Analysis of the indicated proteins in DC2.4 cells that were cocultured with either the Scramble- or Arf1-ablated CT26 cells in the presence or absence of TLR2/6 inhibitors C29 (50 μM) or MMG-11(50 μM). **g, i)** Expression of the indicated genes in DC2.4 cells that were treated as described in (**f**). (n = 3).

Each point represents the percentage of ASC specks (**d**). In all of the panels, data are presented as means ± SEMs; $*p < 0.05$, $**p < 0.01$, $***p < 0.001$, $****p < 0.0001$. n.s., not significant. Student's *t* test.

Supplementary Figure 11

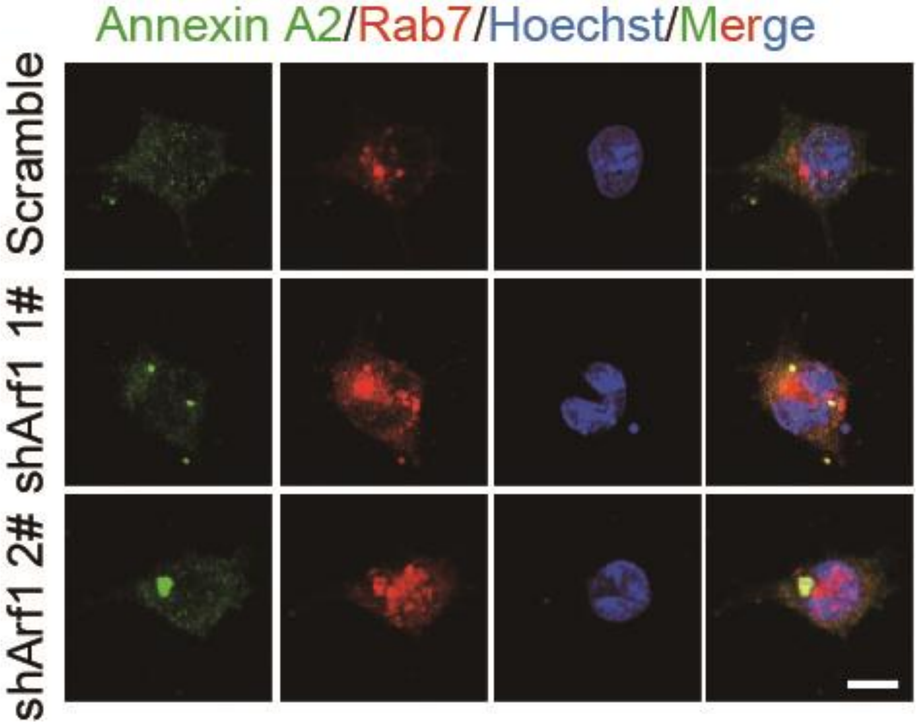

Supplementary Figure 11. The membrane of endosomes was disrupted in DCs after co-culture with Arf1-ablated CT26 cells

Immunofluorescence staining of Rab7 and Annexin A2 in DC2.4 cells after coculture with either Scramble- or Arf1-ablated CT26 cells. Scale bars, 25 µm.

Supplementary Figure 12

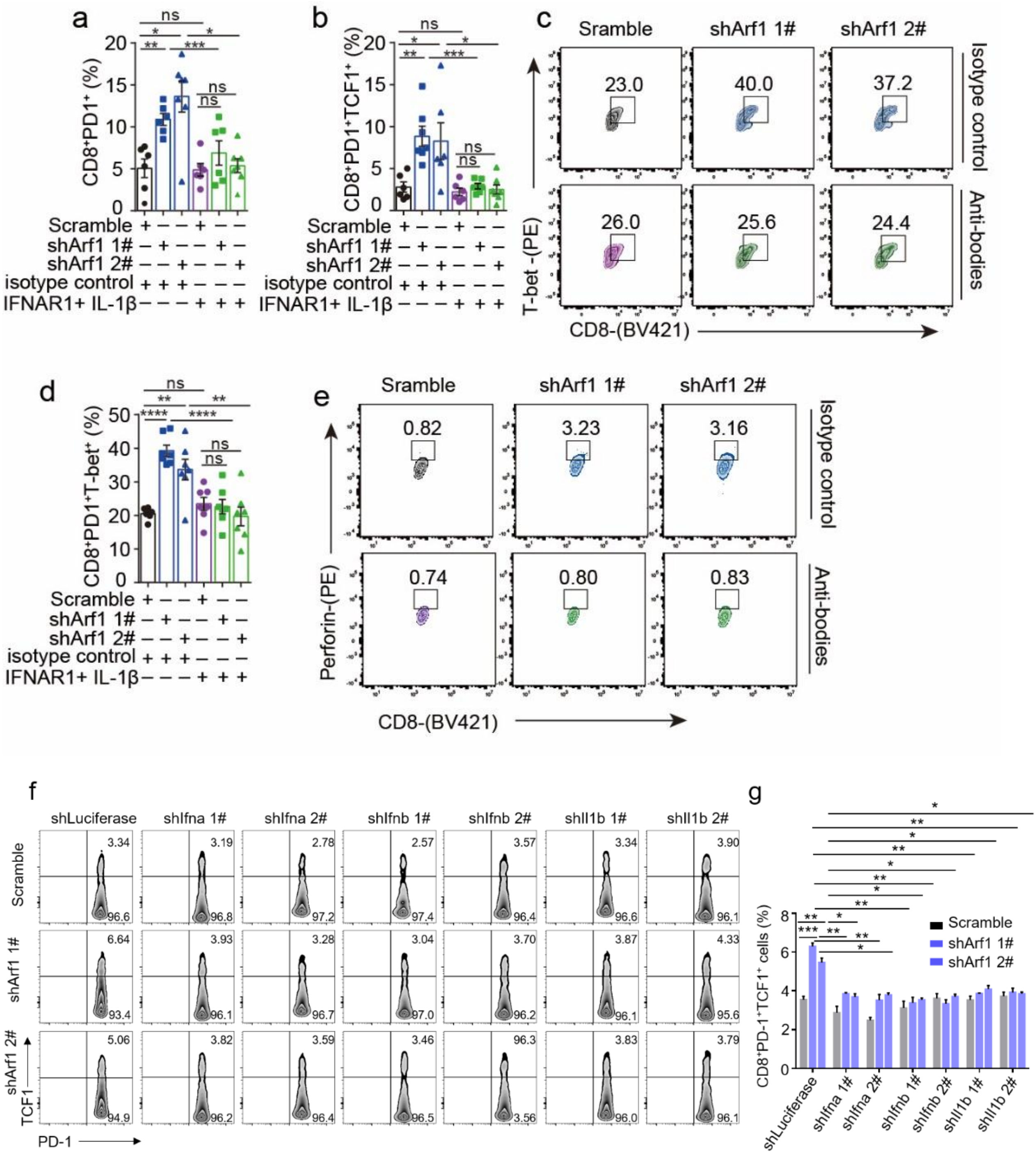

Supplementary Figure 12. The Activated DC-secreted cytokines maintain stem cell-like CD8^{+}T cells

a) Flow cytometry analysis of frequency of the PD1^{+}CD8^{+} T cells in tumors of mice that were transplanted with either the Scramble- or Arf1-ablated CT26 cells and treated

with or without anti-IFNAR1 and anti-IL-1b antibodies (n= 6-7 mice). **b**) Analysis of frequency of the TCF1$^+$PD1$^+$CD8$^+$ T cells in tumors of mice treated as described in (a). (n=6-7 mice). **c, d**) Flow cytometry analysis of T-bet expression (**c**) and quantification of T-bet$^+$ cells (**d**) among the PD1$^+$CD8$^+$ Tumor infiltrating T cells (n= 6-7 mice). **e**) Flow cytometry analysis of perforin expression among the PD1$^+$CD8$^+$ T cells in tumors of mice treated as described in (**a**). (n= 6-7 mice). **f**) Flow cytometry analysis of T cell stemness markers of OT1 T cells cocultured with Il1b and Ifna/b-knockdown dendritic cells activated by Arf1-ablated tumor cells. **g)** quantification of TCF1$^+$PD1$^+$ CD8$^+$ T cells in (f). Two-way ANOVA was used to calculate the P value. *p<0.05, **p<0.01. In panel a-e, data are presented as means ± SEMs; *p < 0.05, **p < 0.01, ***p <0.001, ****p < 0.0001. n.s., not significant. Student's *t* test.
